# Study on CNT/TPU cube under the 3D printing conditions of infill patterns and density

**DOI:** 10.1038/s41598-023-44951-5

**Published:** 2023-10-18

**Authors:** Imjoo Jung, Eun Joo Shin, Sunhee Lee

**Affiliations:** 1 Department of Fashion and Textiles, Dong-A University, Busan, 49315 Republic of Korea; 2 Department of Organic Materials and Polymer Engineering, Dong-A University, Busan, 49315 Republic of Korea; 3 Department of Fashion Design, Dong-A University, Busan, 49315 Republic of Korea

**Keywords:** Materials for devices, Composites

## Abstract

In this study, to develop soft pressure sensor applicable to wearable robots using stretchable polymers and conductive fillers, 3.25 wt% carbon nanotubes/thermoplastic polyurethane filament with shore 94 A were manufactured. Three infill densities (20%, 50%, and 80%) and patterns (zigzag (ZG), triangle (TR), honeycomb (HN)) were applied to print cubes via fused filament fabrication 3D printing. Most suitable infill conditions were confirmed based on the slicing images, morphologies, compressive properties, electrical properties, and electrical heating properties. For each infill pattern, ZG and TR divided the layers into lines and figures, and the layers were stacked by rotation. For HN, the same layers were stacked in a hexagonal pattern. Consequently, TR divided layer in various directions, showed the strongest compressive properties with toughness 1.99 J for of infill density 80%. Especially, the HN became tougher with increased infill density. Also, the HN laminated with the same layer showed excellent electrical properties, with results greater than 14.7 mA. The electrical heating properties confirmed that ZG and HN had the high layer density, which exhibited excellent heating characteristics. Therefore, it was confirmed that performance varies depending on the 3D printing direction, and it was confirmed that HN is suitable for manufacturing soft sensors.

## Introduction

The fused filament fabrication (FFF) method used for 3D printing is a melt-deposition method. For this method, modeling can be implemented by extruding a thermoplastic filament material through a nozzle and stacking layers under various printing conditions^1–3^. The printing conditions were set using a 3D slicing program. Typical printing conditions include the infill pattern, infill density, layer height, print speed, nozzle temperature, and bed temperature, which vary with the material for each 3D slicing program. This value can be set based on the material used. The infill pattern and density can form an internal structure by varying the moving path of the nozzle, and therefore, infill conditions can determine the printing time, filament consumption, and weight and it can affect the physical properties^4–7^. The infill pattern is the most important factor to design the movement of the nozzle and determine the shape and stacking of the layers. Infill patterns can be classified into 2D, 3D, and 3DF (3D flexibility) types. In the 2D type, the nozzle moves and divides the layer into lines in one or two directions and the nozzle moves. Representative 2D-type infill patterns include grids, lines, and zigzags. In the 3D type, the layers are divided into shapes such as triangles and rectangles. Further, the infill patterns of the 3D type include triangle, cubic, cubic subdivision, octet, quarter-cubic, cross, honeycomb, and gyroid. The 3DF types are subdivided into flexible infill patterns (concentric, cross (3D)) that have space, do not have intersections, and have flexible performances among the 3D types^7,8^. Due to infill conditions according to various shapes, research is being conducted on how the formed layer behaves during compression or tension. It was shown that cracking or delamination of layers during compression or tension can affect impact load, strength, elongation, and energy absorption^9–11^. Also, pores in internal structure have unique mechanical properties in some applications and can be used for scaffold design^12,13^. Further, FFF 3D printing can penetrate deeper into internal structures and form cellular structures. The microstructure, orientation, and crystallinity of the polymers were determined based on the nozzle movement path. The microstructural alignment of the filament depends on the nozzle movement path; further, the polymer content differs with the infill density^14–16^.

With recent developments in wearable devices, flexible wearable electrodes have become an attractive field. Wearable application devices include sensors, conductors, actuators, and heater devices^17–20^. Soft sensors have been developed to customize sensors for 3D printing; therefore, soft sensors must be flexible and conductive. Thus, composites with conductive materials and stretchable polymers can be used to provide flexible, soft, stretchable, and conductive properties. In terms of conductivity, carbon nanomaterials such as carbon nanotubes (CNTs), graphene (GR), carbon fiber (CF), carbon black (CB), and crystalline diamond displays are used because of their exceptional electrochemical properties, resulting in their widespread application^21–24^. CNTs composed of six hexagonal graphite sheets connected in a tubular shape exhibit excellent conductivity, mechanical properties, and electrical conductivities. Thermoplastic polyurethane (TPU) is used to impart excellent stretchability and flexibility^25–27^. As for the research on manufacturing sensors by synthesizing TPU and carbon nanomaterials, there have been studies on manufacturing sensors with CNT/TPU^28–30^, GR/TPU^31–34^, and CB/TPU^35,36^. In particular, various studies applying carbon/TPU to the 3D printing process are also being conducted. For example, Li et al.^37^ developed auxetic sinusoidal flexible strain sensors by fused deposition modeling (FDM) 3D printing using TPU, followed by immersion and coating in CNT. The strain sensor had a negative Poisson’s ratio (NPR) and a strain of up to 300%. In addition, the performance was excellent even after 3000 repeated tensile tests. Xiang et al.^38^ prepared CNT/TPU filaments both with and without 1-pyrenecarboxylic acid (PCA) at 1.5 wt% and 3 wt%. The CNT/TPU filament modified with the PCA was better dispersed through its morphology. Compared to the CNT/TPU and modified CNT/TPU, the resistance decreased 37 fold. The sensitivity of the sensor increased with a decrease in CNT content. Therefore, the 1.5 wt% modified CNT/TPU output had the highest tensile elongation at 710% and the second highest tensile strength at 16.5 MPa. In addition, at 250% strain, it was the most detectable under tension at various frequencies. Tang et al.^39^ investigated flexible pressure sensors, which were fabricated by 3D printing using CNTs, fumed silica nanoparticles (SiNPs) as a conductive filler, conductive silicone, and silicone elastomer rubber gel ink. Soft and porous composites (SPCs) exhibit excellent piezoresistive sensitivity and a large linear sensing range. In addition, the sensor was successfully used in grasp-sensing motion and gait-monitoring systems. In particular, in the case of pressure sensors manufactured with an FFF 3D printer, electromechanical property appears different depending on the infill conditions. As mentioned above, how the microstructure and polymer distance along the nozzle's movement path are determined depending on the infill condition during compression is considered a very important research subject^28^.

In our previous study, Choi et al.^40^ fabricated a strain sensor by dip-coating caster-oil-based waterborne polyurethane (CWPU)/GR on four types of auxetic cubes printed with TPU using an FFF 3D printer. Kai et al.^41^ reported the development of a sensor for application in wearable robotic exoskeletons for hands, dip-coated fingertips with three infill densities, and three infill patterns with 8 wt% GR/waterborne polyurethane (WPU). The most suitable infill conditions for soft sensors were determined; however, when the sensor is manufactured using a dip-coating process with a conductive solution, the solution does not completely permeate the 3D-printed (3DP) TPU sample, and the coated surface cracks or peels off during tensile, compression, laundry, abrasion, and wear.

Thus, this study aimed to confirm the most suitable 3D printing infill conditions for developing a conductive soft pressure sensor using a fabricated CNT/TPU filament. CNT/TPU filaments were prepared via synthesis and extrusion; the CNT content was 3.25 wt% and extruded to a diameter of 1.70 mm for use in an FFF 3D printer. The 3DP cube was printed by applying infill conditions with four types of infill densities (0%, 20%, 50%, and 80%) and infill patterns (none (N), zigzag (ZG), triangle (TR), and honeycomb (HN)). The 3DP 3.25 wt% CNT/TPU cubes with various infill densities and patterns were analyzed by surface imaging, morphology, X-ray diffraction (XRD), compressive properties, electrical properties, and electrical heating properties. The polymer orientation of CNT/TPU was predicted according to the infill conditions and the corresponding peroperties were analyzed to confirm the optimal infill conditions.

## Results and discussion

### Morphology of 3.25 wt% CNT/TPU cube with various infill patterns and densities.

**Figure 1:**
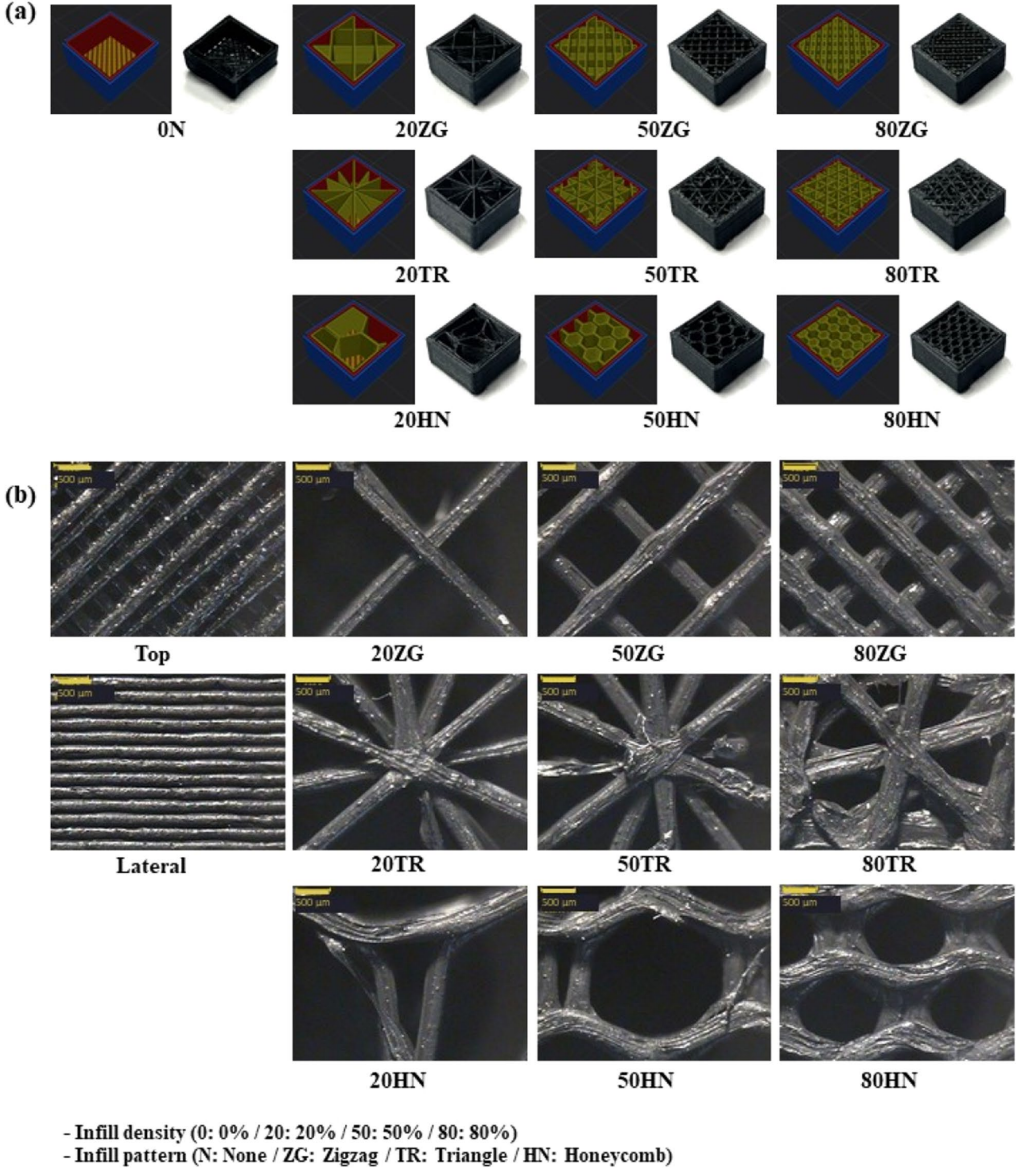
(**a**) Surface slicing images of 3.25 wt% CNT/TPU cube with various infill patterns and densities. (**b**) Morphology of 3.25 wt% CNT/TPU cube with various infill patterns and densities.

Figure 1a shows the surface slicing images of the 3.25 wt% CNT/TPU cube with various infill patterns and densities. At an infill density of 0%, the inner space at 0N was empty. It became denser as the ratio increased from 20 to 80%. The ZG was deposited with layers divided by lines crossing in two directions perpendicular to each other. In the case of TR, the layers were divided into triangles and stacked in the orthogonal direction. In the case of ZG and TR, the line spacing narrowed with an increase in the infill density. HN were deposited using the same hexagonal layers. For HN, the size of the hexagonal units gradually decreased with an increase in the infill density.

The morphologies of 3.25 wt% CNT/TPU cube with various infill patterns and densities are shown in Fig. 1b. In FFF 3D printing, the layers are stacked and constructed, and each layer was partially bonded to the polymeric filaments. The bonding was induced by thermal energy as the filament melted. The bonding phenomenon can be explained by sintering, and the degree of bonding varies depending on the surface tension and viscosity. Further, the bonded part differs based on the infill pattern and density^42,43^. The bonding between the layers was very good for the 3.25 wt% CNT/TPU cube. The TPU exhibited excellent elasticity and adhesion^44^. For TR, the 3D infill type was the most complex. Among the three patterns, HN, which is a 3DF infill type, showed the most porous space.

### Actual time and weight of 3.25 wt% CNT/TPU cube with various infill patterns and densities

**Figure 2:**
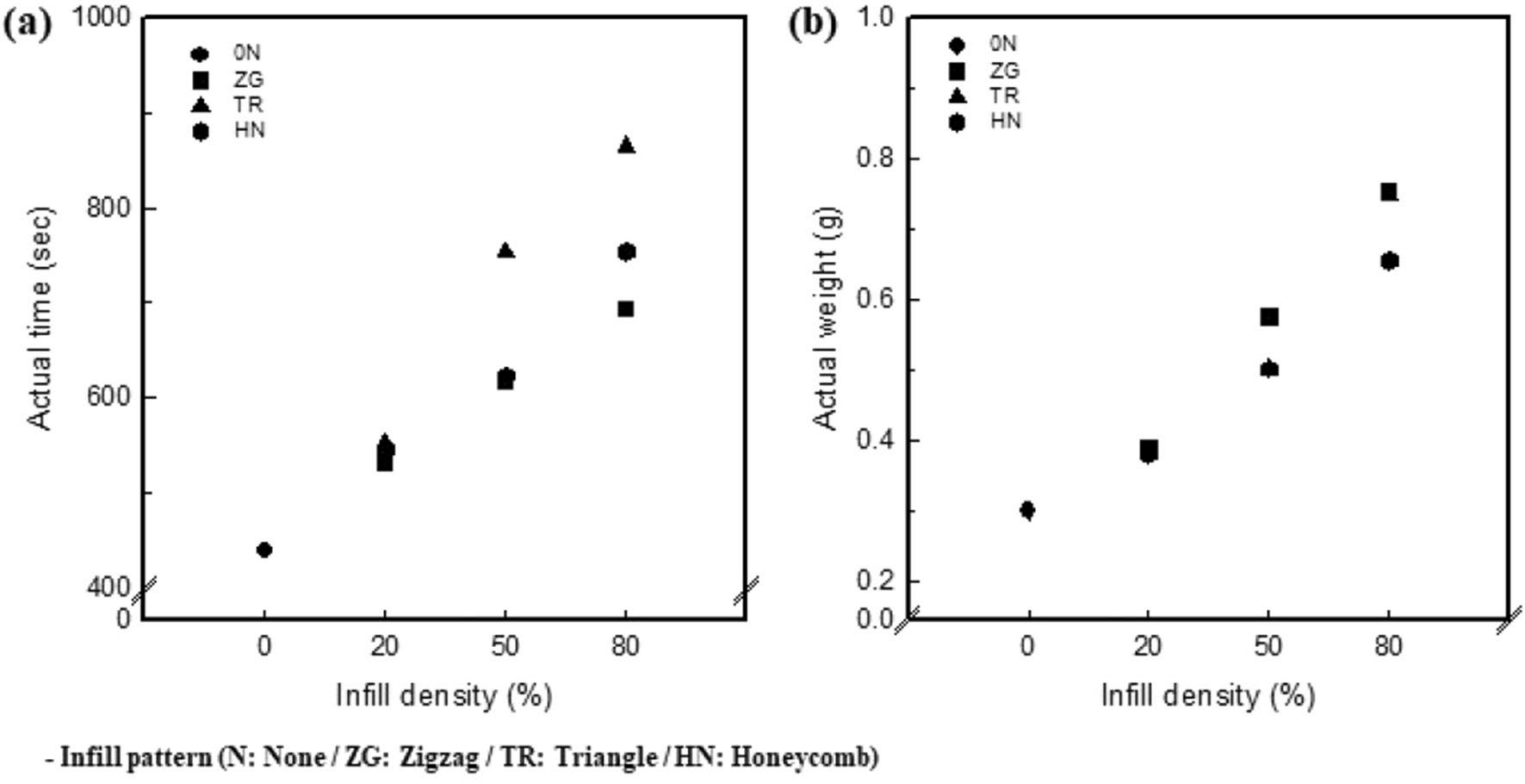
(**a**) Actual printing time of 3.25 wt% CNT/TPU cube with various infill patterns and densities. (**b**) Actual weight of 3.25 wt% CNT/TPU cube with various infill patterns and densities.

Figure 2a and b show the actual printing time and weight of a 3.25 wt% CNT/TPU cube with various infill patterns and densities. For 0 N, the printing time was 7 min 18 s. When the infill density was 20%, the printing times were 8 min 51 s, 9 min 12 s, and 9 min 6 s for 20ZG, 20TR, and 20HN, respectively. At 50% infill density, it took 10 min 17 s, 12 min 34 s, and 10 min 23 s for 50ZG, 50TR, and 50HN, respectively. At 80% infill density, 80ZG, 80TR, and 80HN took 11 min 34 s, 14 m 25 s, and 12 m 34 s, respectively. Printing time was in the order TR > HN > ZG; the time increased with an increase in the infill density. For ZG, the printing time was the shortest because the nozzle path was divided into continuous lines. However, The TR with the longest printing time had the most complicated movement path because the nozzle moved while dividing the layer into triangles. The weights were confirmed to be 0.39 ± 0.01 g, 58 ± 0.01 g, and 0.76 ± 0.05 g for 20ZG, 20TR, and 20HN, respectively, when the infill density is 20%. At an infill density of 50%, 50ZG, 50TR, and 50HN were 0.58 ± 0.01 g, 0.50 ± 0.00 g, and 0.50 ± 0.01 g, respectively. For an infill density of 80%, 80ZG, 80TR, and 80HN were identified as 0.76 ± 0.05 g, 0.75 ± 0.07 g, and 0.65 ± 0.02 g, respectively. The actual weight is larger in the order ZG > TR > HN, and it increases with an increase in infill density. As confirmed by the slicing image and morphology, ZG had the largest weight because of the highest density of the internal space as the lines dividing the layer were located the closest. In contrast, HN had the smallest weight because it had the most space inside.

### Crystallization of 3.25 wt% CNT/TPU cube with various infill patterns and densities

**Figure 3:**
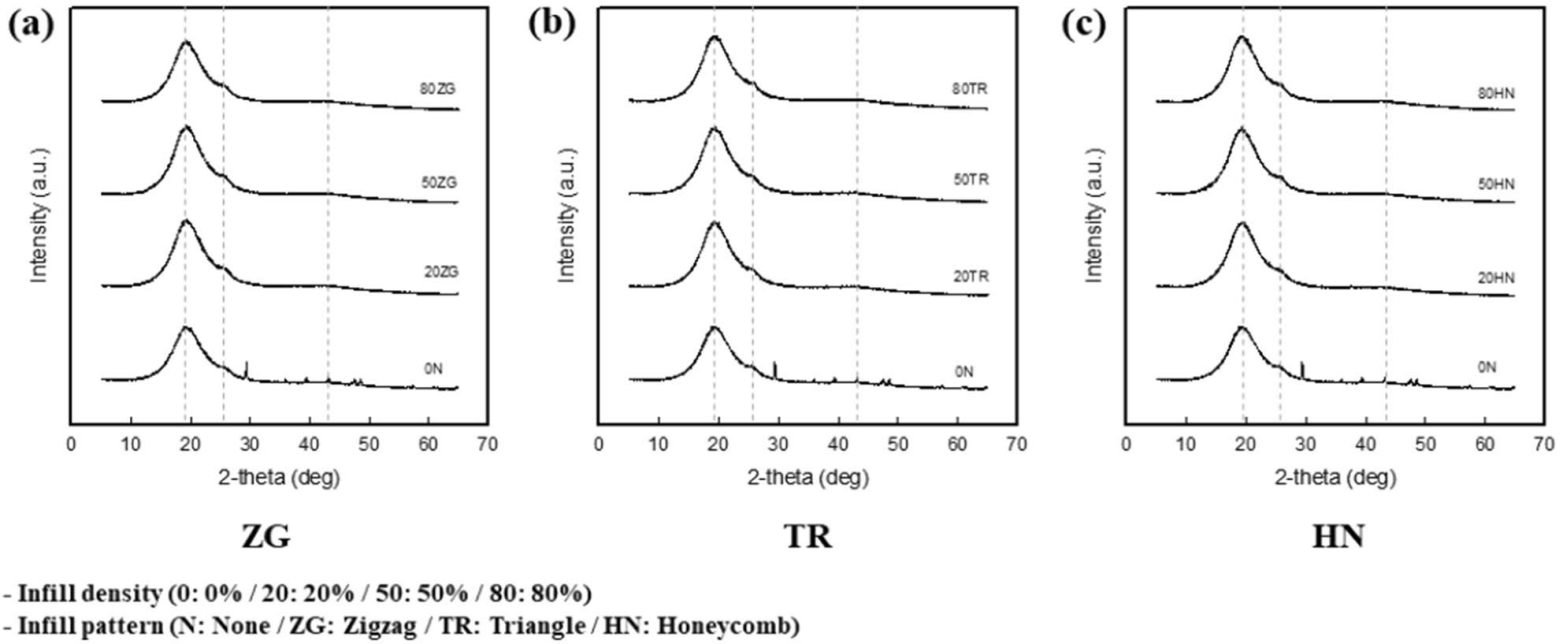
XRD patterns of 3.25 wt% CNT/TPU cube. (**a**) ZG infill pattern with various infill densities, (**b**) TR ZG infill pattern with various infill densities, and (**c**) HN infill pattern with various infill densities.

Figure 3 shows the XRD of 3.25 wt% CNT/TPU with various infill patterns and densities. The results of XRD pattern of the cube with various infill patterns and densities indicated that similar patterns of the cube were presented peak at 2θ = 19.5° and small peak at 2θ = 26.0° and 43.0°. The peak at 2θ = 19.5° is attributed to the influence of TPU. The hard-domain crystalline phase of TPU showed a typical diffraction peak around 2θ = 19.5°, and the amorphous region showed a wide scattering region. Peaks at 2θ = 26.0° and 43.0° appeared because of the effect of the CNT. The peak observed at 2θ = 26.0° originates from the ordered arrangement of the concentric cylinders of graphitic carbon. Peaks around 2θ = 43.0° were attributed to the graphitic plane and small amount of catalyst particles remaining inside the MWCNT walls^45–48^. Therefore, the crystallographic structure of TPU did not vary with the infill conditions.

### Compressive property of 3.25 wt% CNT/TPU cube with various infill patterns and densities

**Figure 4:**
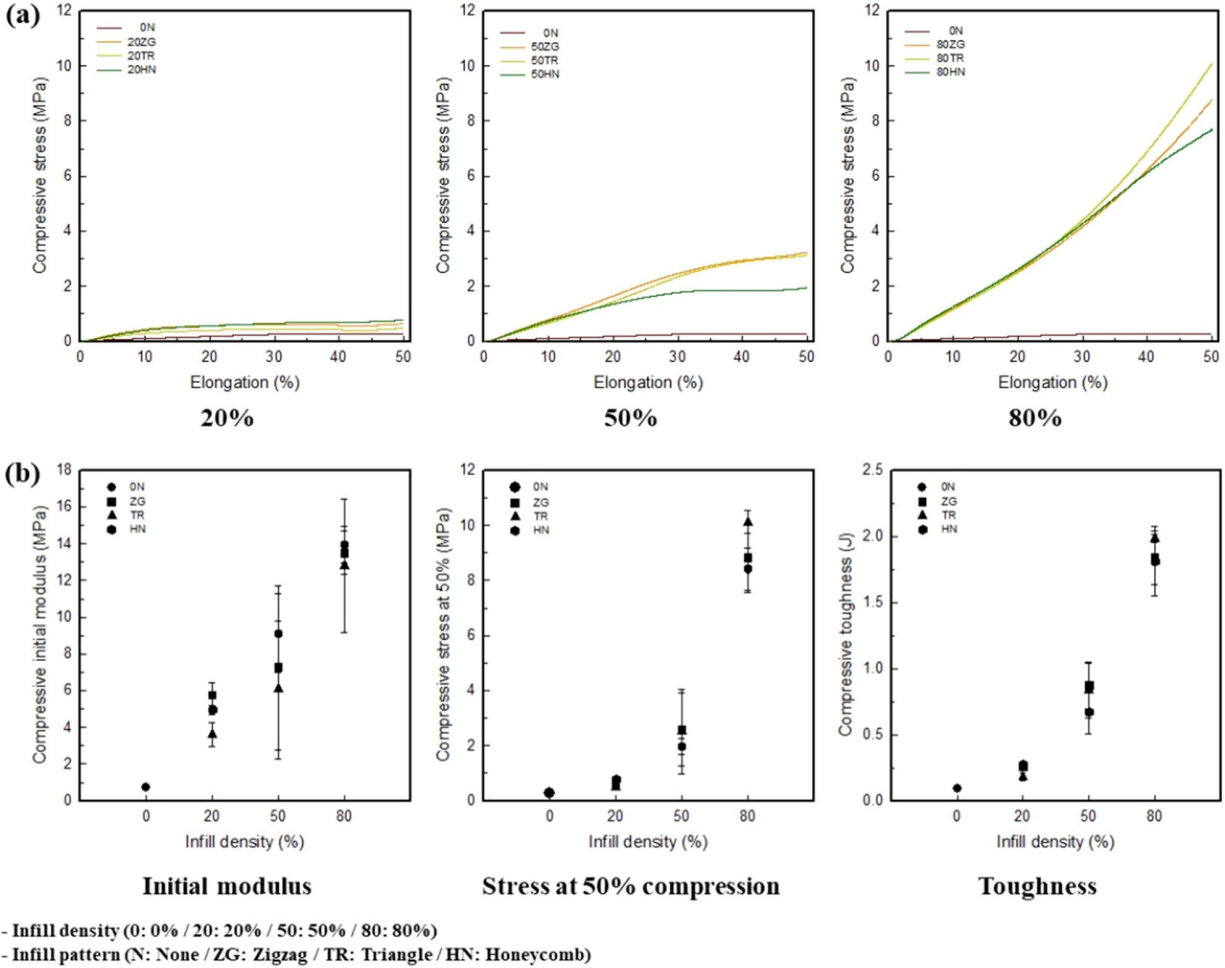
(**a**) Compressive S–S curves of 3.25 wt% CNT/TPU cube with various infill patterns and densities. (**b**) Compressive properties of 3.25 wt% CNT/TPU cube with various infill patterns and densities.

Figure 4a shows the compressive strain–stress (S–S) curves of the 3.25 wt% CNT/TPU cube with various infill patterns and densities. For the 0N sample, the strength was very small and had a yield point of 35%. At an infill density of 20%, a yield point with approximately 30% compressive elongation was observed. This indicates that the initial modulus was superior to that of ZG; however, HN at 50% compression was the best. At an infill density of 50%, ZG exhibited the highest strength, whereas HN exhibits the lowest. The yield point was observed at a compressive elongation of approximately 35%. No yield point was observed at an infill density of 80%; the strength of TR was the highest. The compressive strengths of the samples increased with the infill density.

Figure 4b shows the compressive property of the 3.25 wt% CNT/TPU cube with various infill patterns and densities. The initial moduli were 5.76, 9.13, and 13.94 MPa for 20ZG, HN, and 80HN, respectively. They were 3.61, 6.06, and 12.79 MPa for 20TR, 50TR, and 80TR, respectively. Thus, the highest compressive property for ZG was at 20% infill density, and the highest for HN was at 50% and 80% infill densities. The TR pattern exhibited the lowest initial modulus. At 50% compression, the compressive stresses were 0.79, 2.59, and 10.12 MPa for 20HN, 50ZG, and 80TR, respectively. For toughness, 0 was 0.09 J and ZG was 0.26 J, 0.87 J, and 1.84 J; TR was 0.18 J, 0.84 J, and 1.99 J; and HN was 0.27 J, 0.67 J, and 1.81 J, as infill density increased from 0 to 80%. Therefore, the strength of the sample increased with an increase in the infill density. For the infill pattern, TR exhibited the best performance, and HN was the toughest. In the case of TR, the rate of increase in the compressive strength was the greatest with an increase in the infill density. During the FFF process, the movement path of the nozzle was determined based on the infill conditions. The movement path of the nozzle determines the internal shape and affects the physical properties^42,43,49^. Therefore, as confirmed by the slicing image and morphology, the TR pattern, which was stacked with the most complex layers. And it showed more layer-bonded parts that were generated by intersection than those of ZG and HN. Therefore, the compressive stress was strongest at 50% and 80% infill due to the large number of layer-bonded parts during compression. On the other hand, HN had the strongest compressive stress at 20% infill density, but as the infill density increased to 50% and 80%, ductile and elastic regions were confirmed at about 30% and 40% compressive strain. As it can be considered that in the slicing-images and morphology, it was porous inside compared to ZG and TR. Thus, it showed the most elastic and toughest performance with an increase in the infill density. According to results, it was indicated that HN is most suitable for use as a soft pressure sensor.

### Electrical conductivity property of 3.25 wt% CNT/TPU cube with various infill patterns and densities

**Figure 5:**
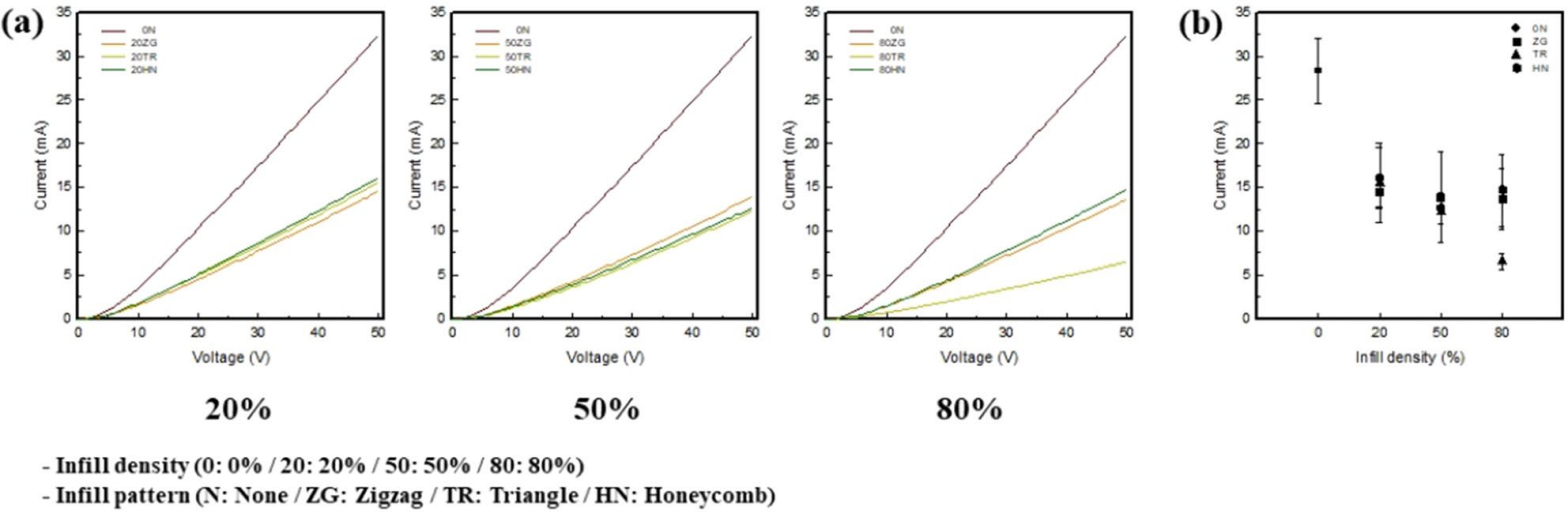
(**a**) Electrical I–V curves of 3.25 wt% CNT/TPU cube with various infill patterns and densities and (**b**) current at 50 V of 3.25 wt% CNT/TPU cube with various infill patterns and densities.

Figure 5 shows the electrical properties of the 3.25 wt% CNT/TPU cube with various infill patterns and densities. For the 0 N sample, the current value was more than twice as large as the others at 28.27 mA. The current values of 20% infill density was 14.50, 15.52, and 16.09 mA for 20ZG, 20TR, and 20HN. For 50% infill density, the current values at 50 V are 13.87, 12.23, and 12.63 mA for 50ZG, 50TR, and 50HN, respectively. For an 80% infill density, the values of current at 50 V were 13.63, 6.48, and 14.70 mA for 80ZG, 80TR, and 80HN, respectively. The current tended to decrease with an increase in the infill density.

For the infill pattern at each infill density, 20HN, 50ZG, and 80HN showed the highest conductivities at 16.09 mA, 13.87 mA, and 14.7 mA, respectively. The conductivity of HN had excellent electrical property because regular hexagonal layers were stacked in the same layer. In contrast, the conductivity of TR decreased rapidly with an increase in infill density. Electrical properties can be analyzed in terms of the slicing image and morphology. Conductivity decreases with an increase in the length of the sample. Further, conductivity is proportional to length and inversely proportional to the cross-sectional area, and therefore, conductivity decreases with an increase in length^50^. The moving path of the output nozzle also increased with an increase in infill density. In addition, the nozzle movement path increased and the printing time was the longest. If there were many moving paths of the nozzle, the path through which the current flowed was the longest; the conductivity decreased. In conclusion, TR with the most complex infill pattern showed the longest printing time and lowest conductivity. Thus, it was confirmed that HN, in which the same layers were stacked so that the current flow most uniformly, had the best conductivity and was suitable for use as a pressure sensor.

### Electrical heating property of 3.25 wt% CNT/TPU cube with various infill patterns and densities

**Figure 6:**
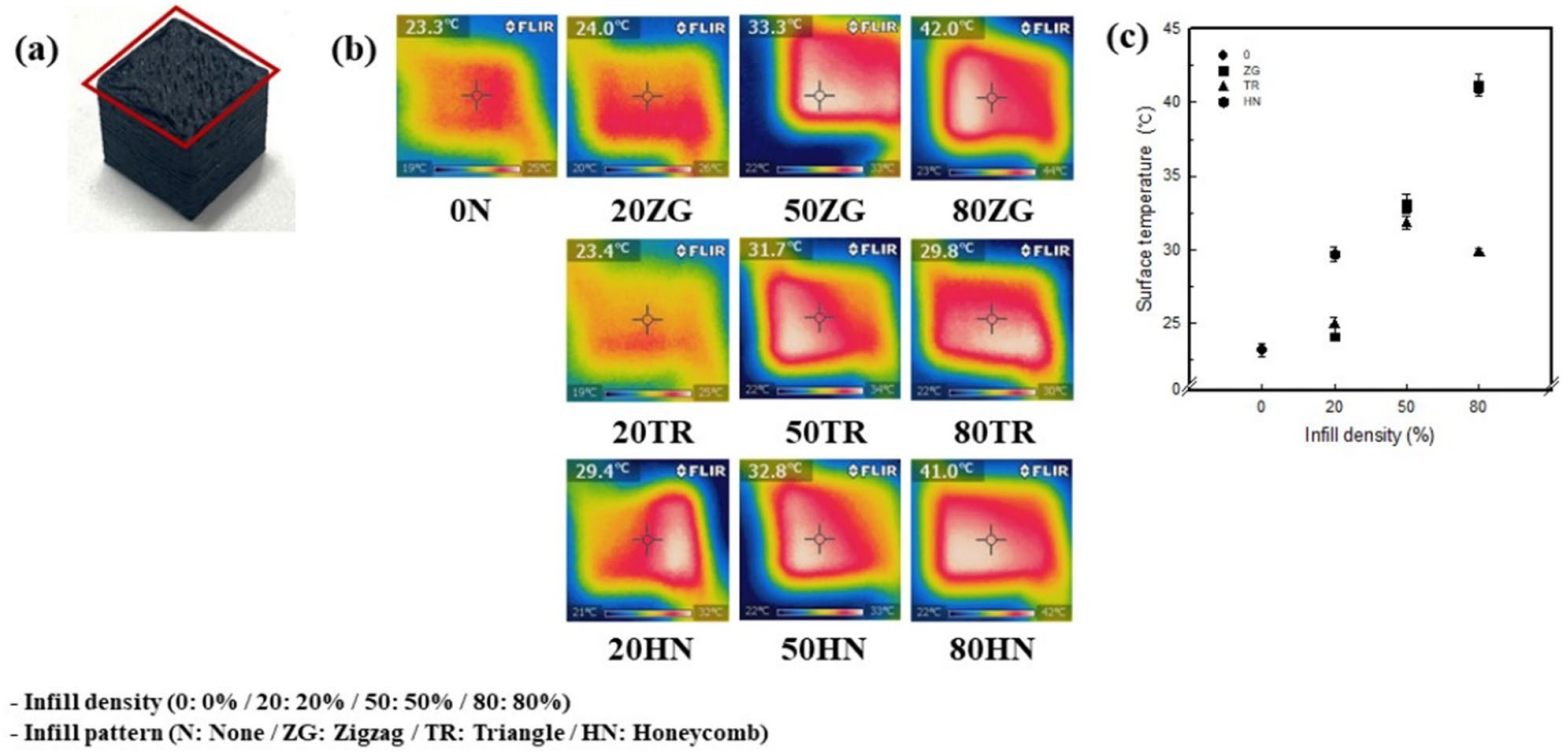
(**a**) Measurement location of electric heating applied 50 V for 5 min of 3.25 wt% CNT/TPU cube with various infill patterns and densities. (**b**) Image of surface temperature applied 50 V for 1 min of 3.25 wt% CNT/TPU cube with various infill patterns and densities. (**c**) Surface temperature applied 50 V for 1 min.

Figure 6 shows the electrical heating properties of the 3.25 wt% CNT/TPU cube with various infill patterns and densities. When 50 V was applied for 5 min, the surface temperature of 0 N was measured at 23.2 ℃. For a 20% infill density, the surface temperatures were 24.1, 24.9, and 29.7 ℃ for 20ZG, 20TR, and 20HN, respectively. The surface temperatures for a 50% infill density were 33.1, 31.8, and 32.8 ℃ for 50ZG, 50TR, and 50HN, respectively. For the 80% infill density, they were 41.2, 29.8, and 40.9 ℃ for 80ZG, 80TR, and 80HN, respectively.

The heating temperatures were high and in the order 80% > 50% > 20% > 0%. The higher the infill density, the higher were the electrical heating temperatures. 20HN at a 20% infill density and 50ZG and 80ZG at 50% and 80% infill densities have the highest electrical heating temperatures of 29.7 ℃, 33.1 ℃, and 41.2 ℃. The exothermic temperature increased with an increase in the infill density. The electric heating property had the highest temperature in the 80% infill density and the ZG infill pattern with the highest internal density was attributed to the influence of the slicing image and morphology. In contrast, the conductivity of 80TR decreased rapidly, and therefore, no heat was generated. In addition, with an increase in the infill density, there was a decrease in conductivity and an increase in surface temperature; this was confirmed by resistance heating. Resistance heating can be confirmed by the increase in the CNT content with an increase in the infill density. When a voltage is applied, current flows in the CNT portion, and electrons move and change in an orderly manner and collide with each other, resulting in heat generation^50,51^. Additionally, 41–42 ℃ in 80ZG and 80HN were found to be very suitable as sensors for wearable robots. It was confirmed that when heaters operate at temperatures above 44 ℃, skin burn and tissue injury can occur^52,53^. Therefore, the pressure sensor in this study has appropriate electrical heating performance because it is heated to a temperature of 44 ℃ or less even when voltage is applied for 5 min.

### 3DP CNT/TPU orientation models with various infill patterns and densities

**Figure 7:**
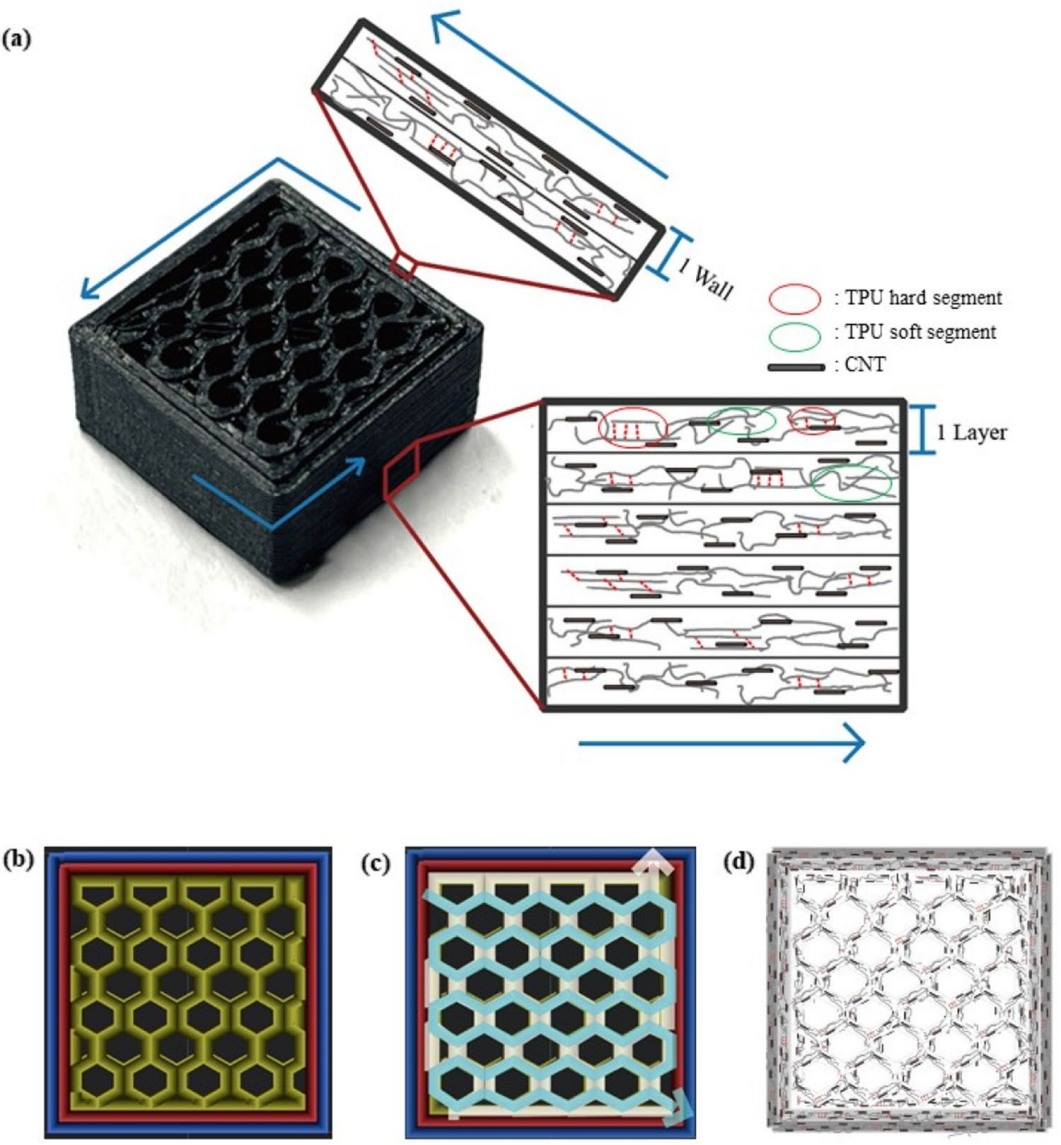
(**a**) Orientation model of wall and lateral layer of 3.25 wt% CNT/TPU polymers, (**b**) Slicing image, (**c**) Nozzle movement path, and (**d**) Schematic of the CNT/TPU orientation of 80HN.

Figure 7a shows the 3.25 wt% CNT/TPU orientation model of the outer walls and cube lateral side. Figure 7b–d show a CNT/TPU slicing image with an infill density of 80%, the nozzle movement path, and a schematic of the CNT/TPU orientation among the HN infill patterns with the best performance in this study. The orientations of the CNT and TPU were stacked in an identical manner to the morphology based on the moving path of the nozzle. The electrical and electrical-heating characteristics can be understood based on an orientation diagram. The same layers were successively deposited for HN. The CNTs were placed in the same direction when each part was viewed in detail; thus, when voltage was applied, electricity could flow without being significantly hindered. The nozzle movements and schematics of different fill patterns and fill densities are shown in Supplementary Table S1 in Supplementary information. ZG had a higher density than the other patterns, and in the case of TR, the obstacles to electrical flow increased because of its complex structure. Therefore, manufacturing a soft sensor HN in which the same layers are stacked can be considered the most suitable infill pattern. In addition, the CNT content increased with increasing density.

## Conclusions

A 3.25 wt% CNT/TPU filament was manufactured, and a cube was printed under various infill patterns and density conditions to confirm the surface images, X-ray diffraction, compressive properties, electrical properties, and electrical heating properties. As results of the actual time and weight, the actual time increased in the order TR > HN > ZG, and the time increased with an increase in the infill density. The weight increased in the order ZG > TR > HN with an increase in the infill density. ZG was confirmed to have the highest internal density due to its short output time and greatest weight. For results of XRD, peaks of TPU and CNT were confirmed at 2θ = 19.5°, 25.5°, and 43.0°. Depending on the infill density and pattern, the sizes of the peaks did not show large differences, confirming that the crystal structures were similar. In the case of the compressive property, the initial modulus was 5.76 MPa, 9.13 MPa, and 13.94 MPa for 20ZG, 50HN, and 80HN, respectively. While the TR was the smallest under all infill density conditions. However, in terms of max stress and toughness, 20HN, 50ZG, and 80TR were 0.27 J, 0.87 J, and 1.99 J, respectively. The compressive stress of the CNT/TPU cubes increased with infill density. Although TR was the hardest, HN exhibited tough performance with an increase in infill density. In case of electrical property, the current increased with an increase in the applied voltage. But the current decreases with an increase in density as internal current length increased. HN was at 16.09–14.7 mA from infill density 20–80%; conductivity was the best. In addition, for the electrical heating properties, the temperature increased with an increase in the infill density, and ZG and HN exhibited the high temperature. Therefore, it was confirmed that the internal shape affected all properties. Further, HN, in which regular hexagonal layers are stacked in the same layer, was suitable as an infill condition for soft pressure sensors with excellent compressive, electrical, and electrical heating properties. In addition, this study is expected to be used as basic data when manufacturing CNT/TPU pressure sensors using a 3D printer.

## Methods

### Materials

In this study, carbon nanotubes (CNT; Carbon Nanomaterial Technology Co. Ltd., Korea) were used as electrical fillers. To manufacture thermoplastic polyurethane (TPU) as a stretchable polymer, polyphenylene sulfide (PPS, Donga Chemical, Korea), 1,3-Propanediol (PDO, Susterra ®, Dupont, USA), 4,4′-methylene dicyclohexyl diisocyanate (H_12_MDI, Vestanat ®, Germany), and tin (II) 2-ethylhexanoate (T_9_, Sigma-Aldrich, USA) were used.

### Preparation of CNT/TPU filament

**Figure 8:**
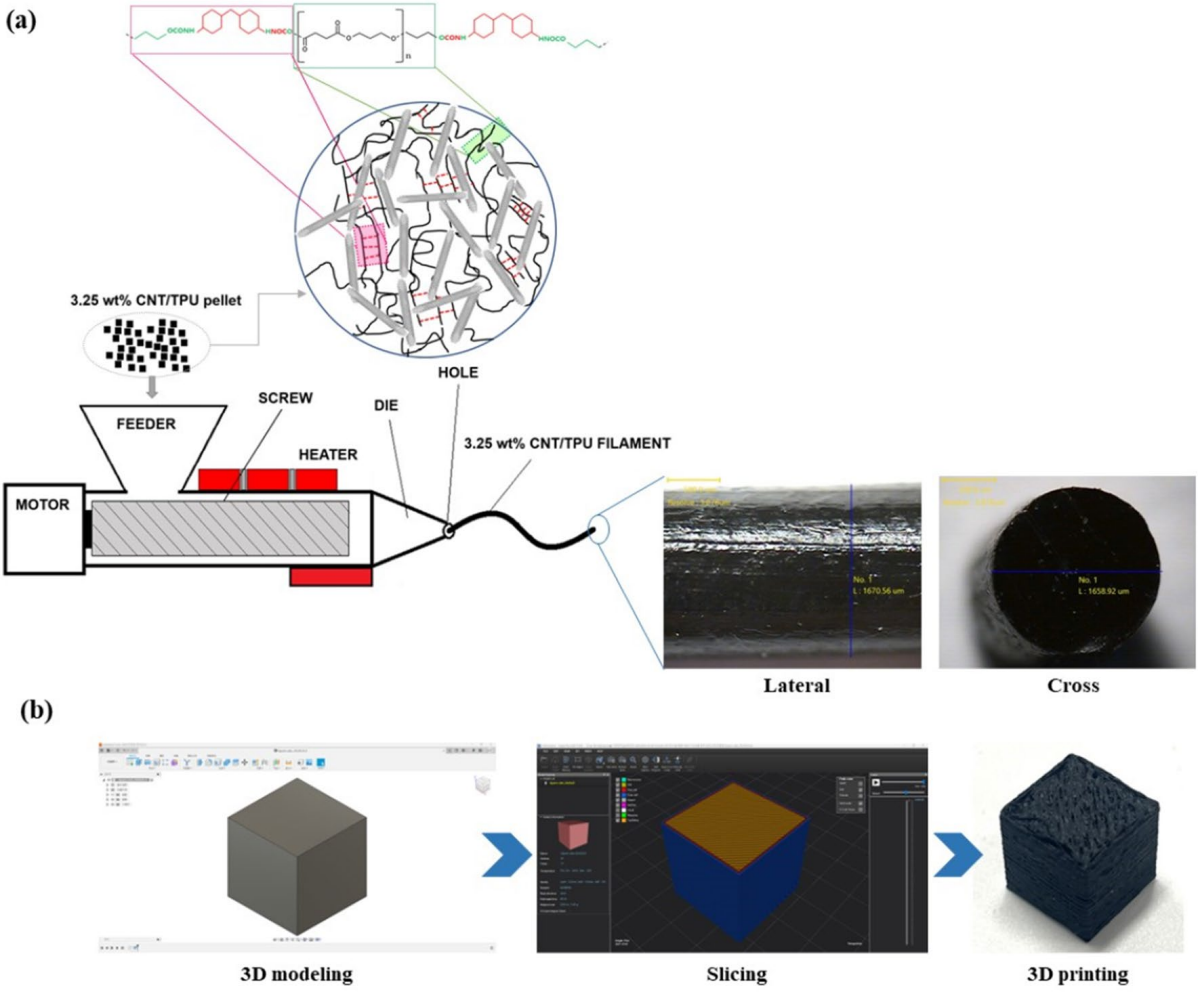
(**a**) Scheme for manufacturing 3.25 wt% CNT/TPU filament and (**b**) 3D printing process.

Figure 8a shows the scheme for manufacturing a 3.25 wt% CNT/TPU filament. The CNT/TPU was synthesized in situ to prepare CNT/TPU pellets. 3.25 wt% CNT was added to the PPS and stirred at 2000 rpm for 5 min using a Thinky Mixer (GR-8, THINKY Corporation, China). Thereafter, PDO was added and dried in a dryer at 100 ℃ for 30 min, and then, H_12_MDI was added. T_9_ was added as a catalyst and stirred at 1200 rpm for 15 min. The molar ratio of the OH groups of the PPS, NCO groups of the H_12_MDI, and OH groups PDO was set as [OH]:[NCO]:[OH] = 1.0:2.7:1.7; the synthesized TPU was dried in a vacuum dryer at 80 ℃ for 20 h. The synthesized 3.25 wt% CNT/TPU was produced in pellets, which were extruded at 3.3 rpm at 240 ℃ with Philibot H400 (Fordentech, Korea) composed of a Shore 94 A filament with a diameter of 1.70 mm.

### Fabrication of 3DP CNT/TPU cube

Figure 8b shows the 3D printing process of the 3.25 wt% CNT/TPU cube. To fabricate the 3.25 wt% CNT/TPU cube, 3D modeling was conducted using the Fusion 360 modeling program (Autodesk, USA). The size of the 3.25 wt% CNT/TPU was designed to be 10 × 10 × 10 mm^3^. Then, the designed 3.25 wt% CNT/TPU cube was 3DP under various infill conditions using an FFF printer (Cubicon single plus, Cubicon Co. Ltd., Korea). 3DP conditions were set using a slicing program (Cubicreator v4.4; Cubion Co., Ltd., Korea). The infill densities were set to 0%, 20%, 50%, and 80%, and the infill patterns were set to zigzag, triangular, and honeycomb. The nozzle temperature, bed temperature, and printing speed were 260 ℃, room temperature, and 60 mm/s, respectively. Table 1 indicates the sample codes of 3DP 3.25 wt% CNT/TPU cube.

**Table 1:** Sample code and specifications of the 3.25 wt% CNT/TPU cube.

Sample code	Infill density (%)	Infill pattern
0N	0	None
20ZG	20	Zigzag
20TR		Triangle
20HN		Honeycomb
50ZG	50	Zigzag
50TR		Triangle
50HN		Honeycomb
80ZG	80	Zigzag
80TR		Triangle
80HN		Honeycomb

### Characterizations

3DP 3.25 wt% CNT/TPU cubes were analyzed in terms of the slicing image, morphology, actual printing time and weight, X-ray diffraction (XRD), compressive properties, electrical properties, and electrical heating properties. Infill conditions inside the 3DP 3.25 wt% CNT/TPU cube under various infill conditions were confirmed by slicing the images and morphologies. The sliced images were viewed on the output preparation screen of the slicing program. The path type was set to speed, and the model was confirmed to be solid. 50% 3D printing was performed and compared to the slicing program. The morphology was examined at 4.5× magnification of the output in a state where only 50% of the output was printed. A fabric image analysis system (NexMeasure Pro5 NTZ-6000, Bestec Vision Co. Ltd.) was also used. After 3D printing, the actual printing time and weight were determined. The actual weight was measured using an electronic balance (PAG114, OHAUS, USA). Crystalline structures of the 3DP 3.25 wt% CNT/TPU cube were confirmed by XRD using an X-ray diffractometer (EMPYREAN, Malvern Panalytical, United Kingdom) under Ni-filtered CuKa radiation. The analysis was performed in the measurement range of 5°–90°. To confirm the compressive properties of the 3DP, 3.25 wt% CNT/TPU was measured using the KS M ISO 604 standard. Experiments were implemented using a universal testing machine (AGS-X, Shimadzu, Japan) with a 5 kN load cell; it was compressed in the z-direction at a speed of 10 mm/min, and the maximum stroke rate was 50%. The compressive S–S curve, compressive initial modulus, compressive stress at 50%, and compressive toughness were obtained and analyzed. The electrical properties of the 3DP 3.25 wt% CNT/TPU cube material were measured using a 2450 source meter (KEITHLEY, Washington, DC, USA) based on KS C IEC 62624. The voltage was applied in the range of 0–50 V at intervals of 0.5 V. The current (A) was checked at the applied voltage. The surface temperature was measured to investigate the electrical heating properties of the 3DP 3.25 wt% CNT/TPU cube. The surface temperature was measured using a 2450 Source Meter (KEITHLEY, USA) and thermal imaging camera (FLIR i5, FLIR Systems Inc., USA). Similar to the time-dependent temperature changes, a cube voltage was applied to the cube at 50 V for 5 min. The temperatures of the upper surfaces of the cubes were measured.

### Supplementary Information

Supplementary Table S1.

## Data Availability

The datasets used and/or analyzed during the current study available from the corresponding author S. Lee (shlee014@dau.ac.kr) on reasonable request.
