# Supplementary material for: Study on CNT/TPU cube under the 3D printing conditions of infill patterns and density
Source: Sci Rep. 2023 Oct 18;13:17728. doi: 10.1038/s41598-023-44951-5 (PMC10584865; doi:10.1038/s41598-023-44951-5)
Supplement: Supplementary file 1 — Supplementary Table S1. [file 41598_2023_44951_MOESM1_ESM.docx]

**Supplementary materials**

**Table S1**. Schematic diagram of CNT orientation of 3.25 wt% CNT/TPU cubic with various infill patterns and densities

| **Sample** | **Path** | **1 layer** | **2 layer** | **1+2 layer** |
| --- | --- | --- | --- | --- |
| **20ZG** | **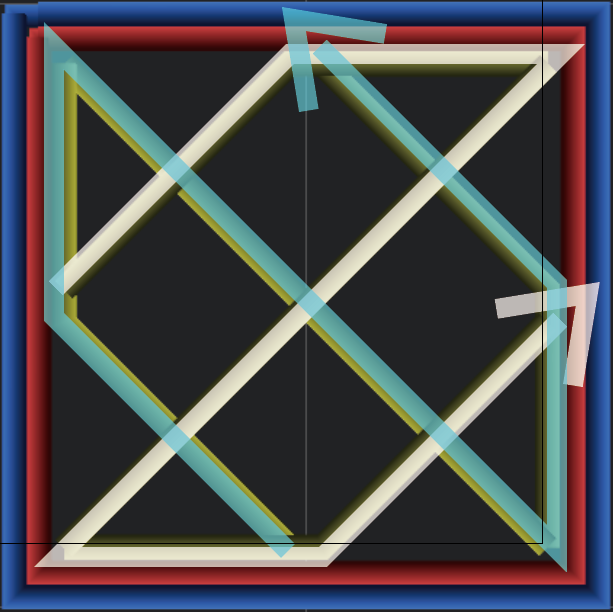** | **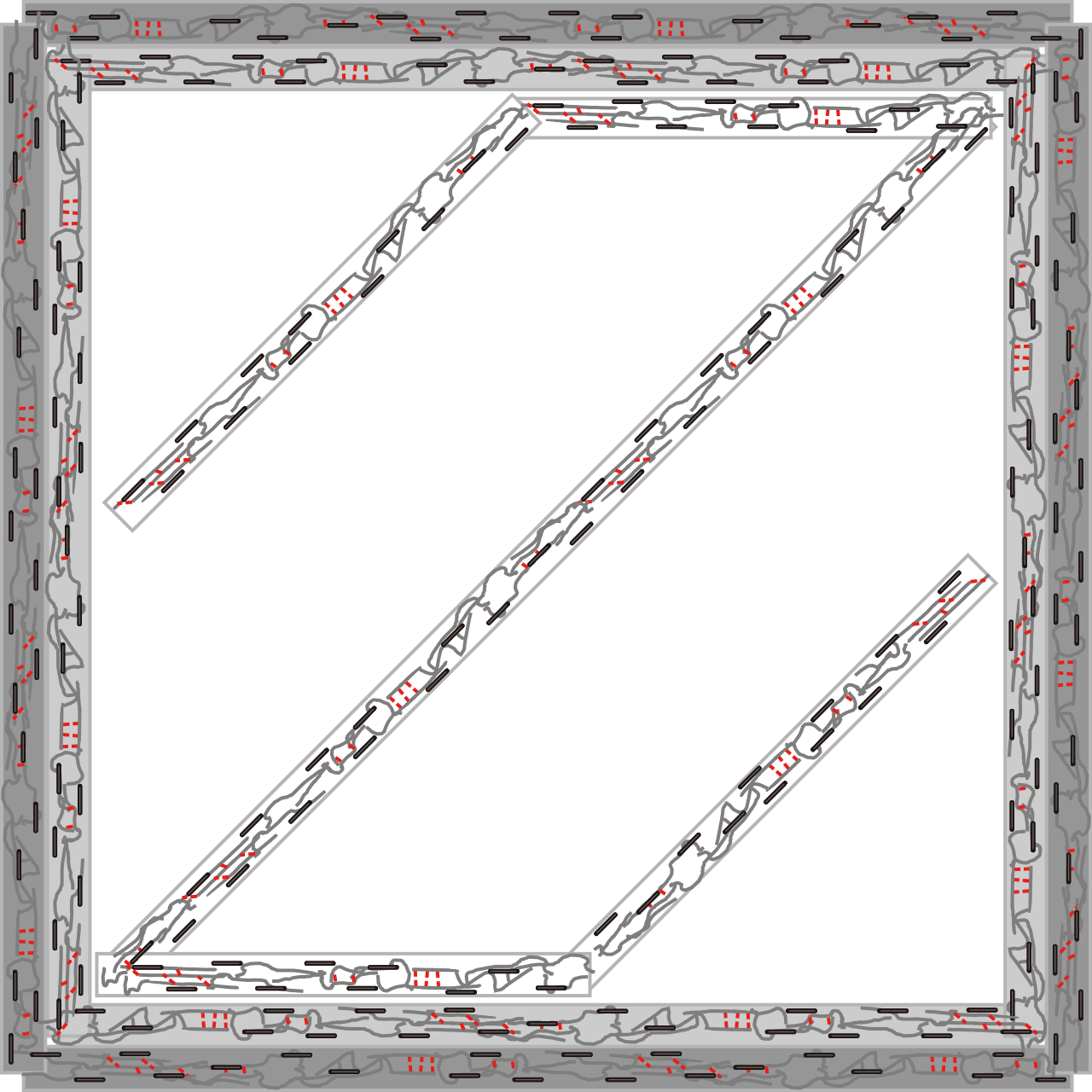** | **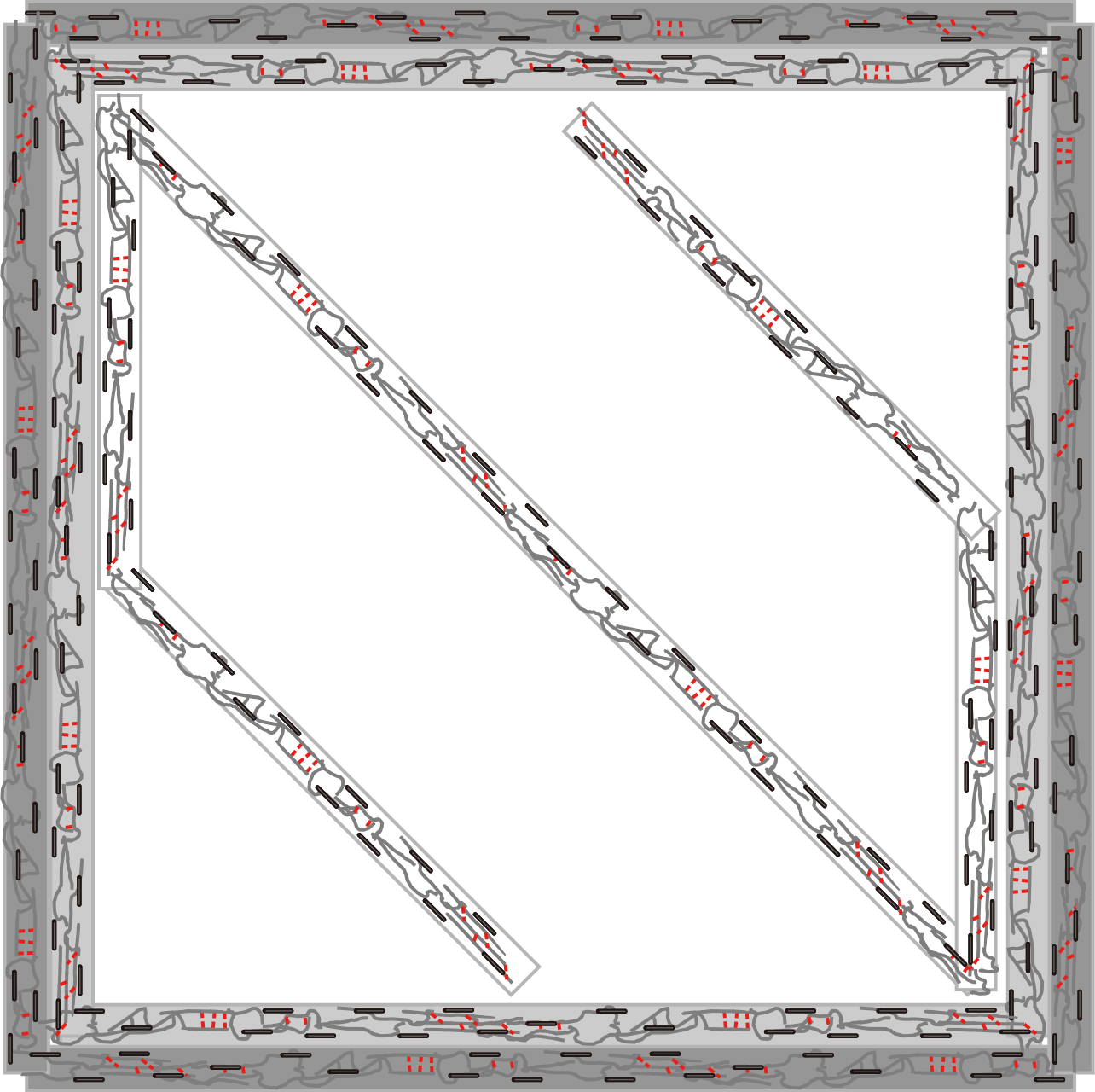** | 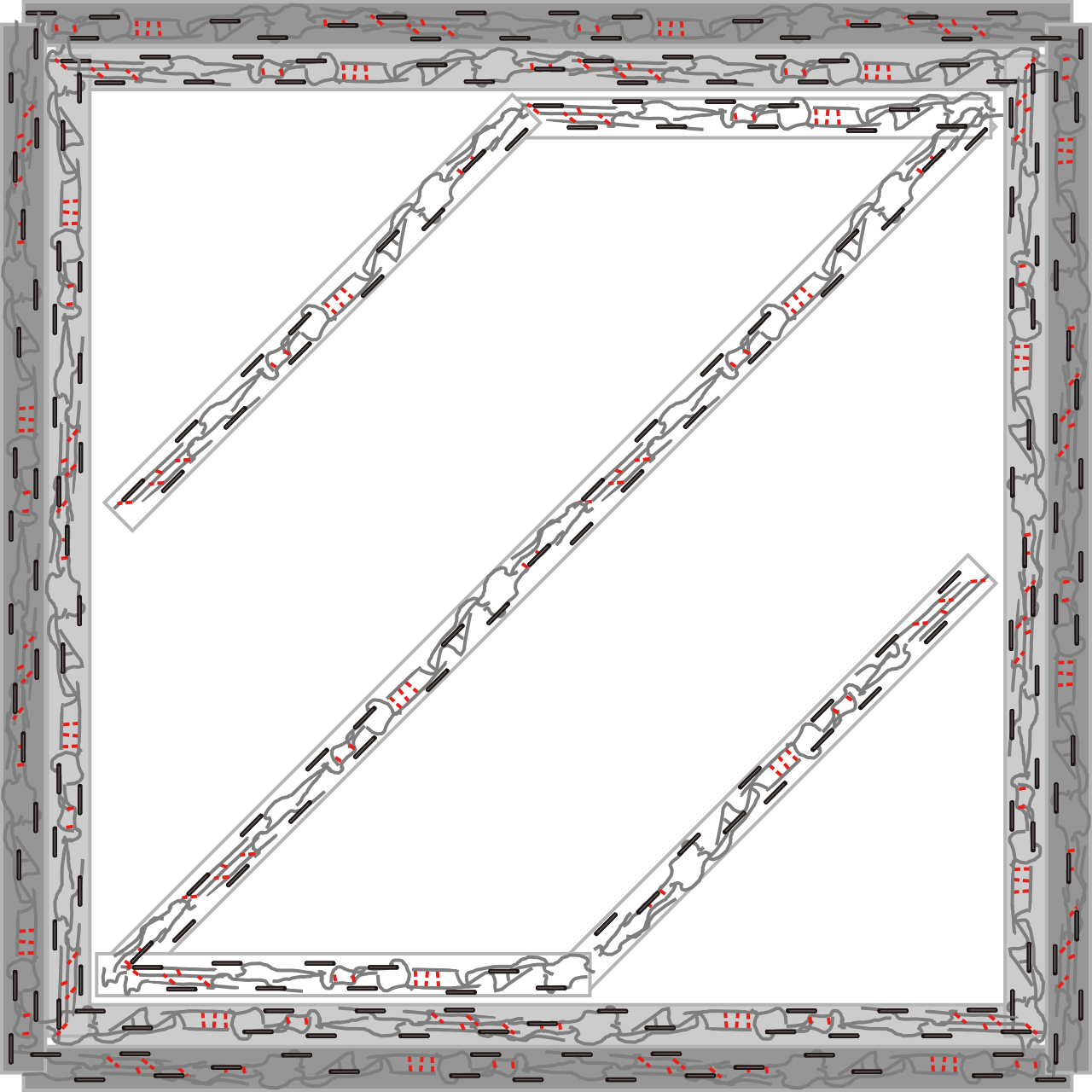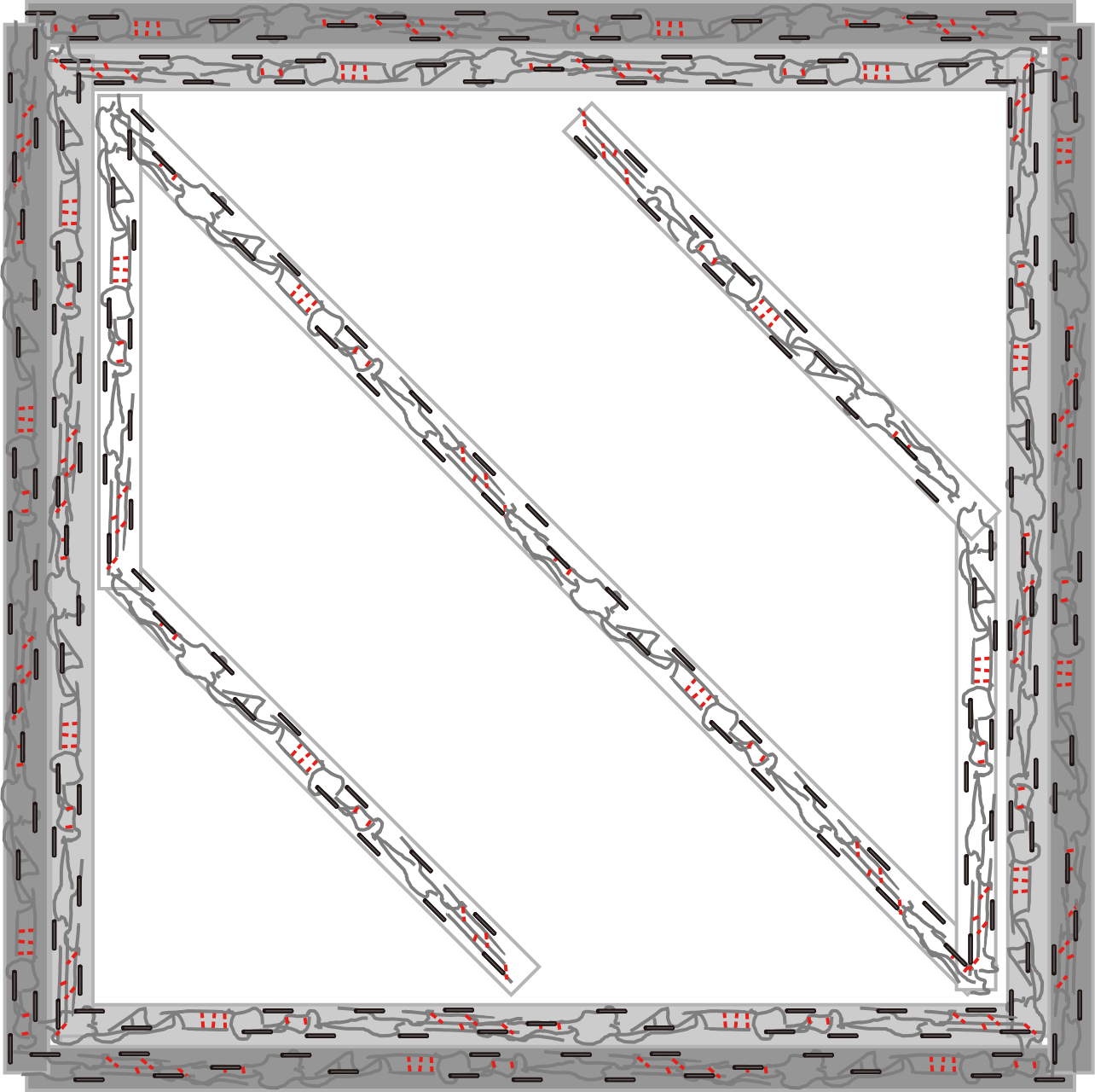 |
| **50ZG** | **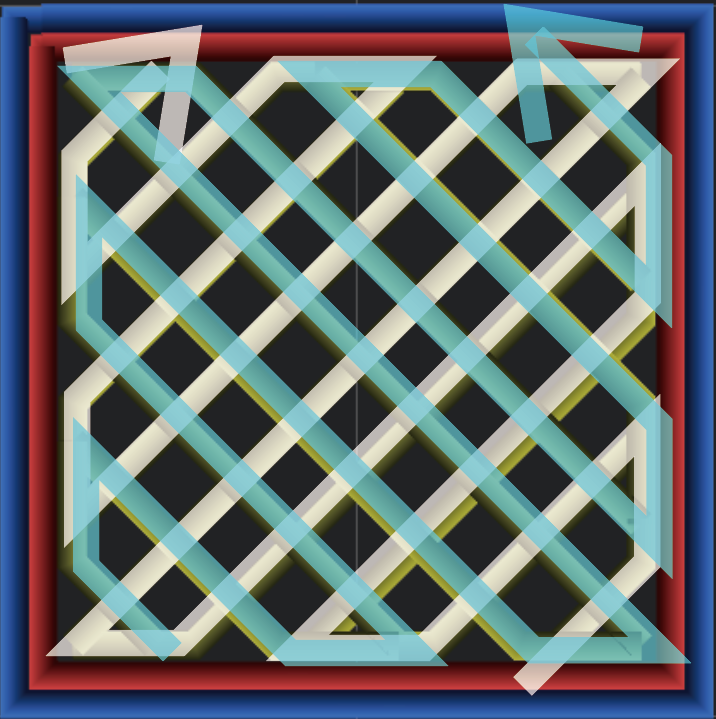** | **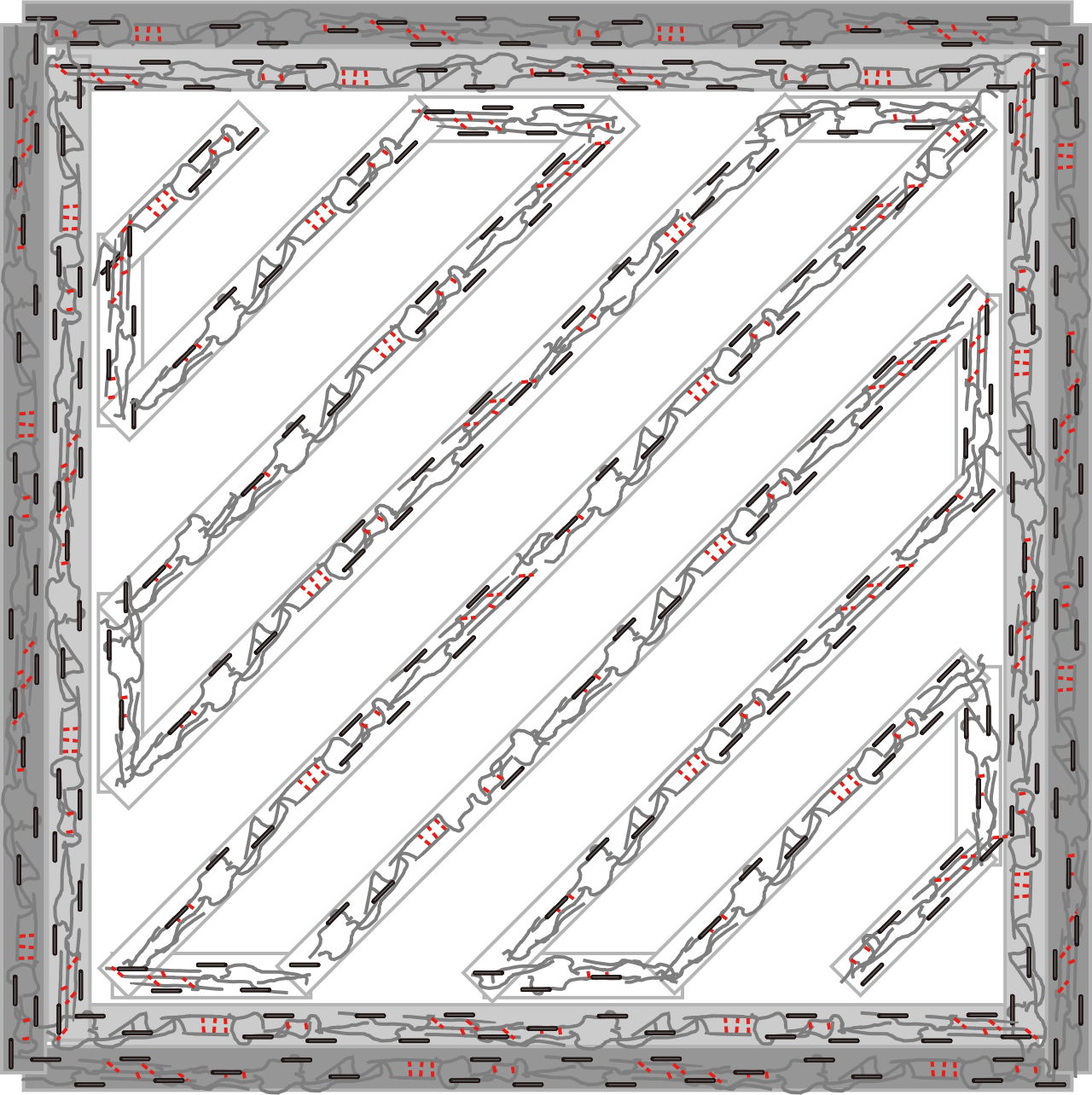** | **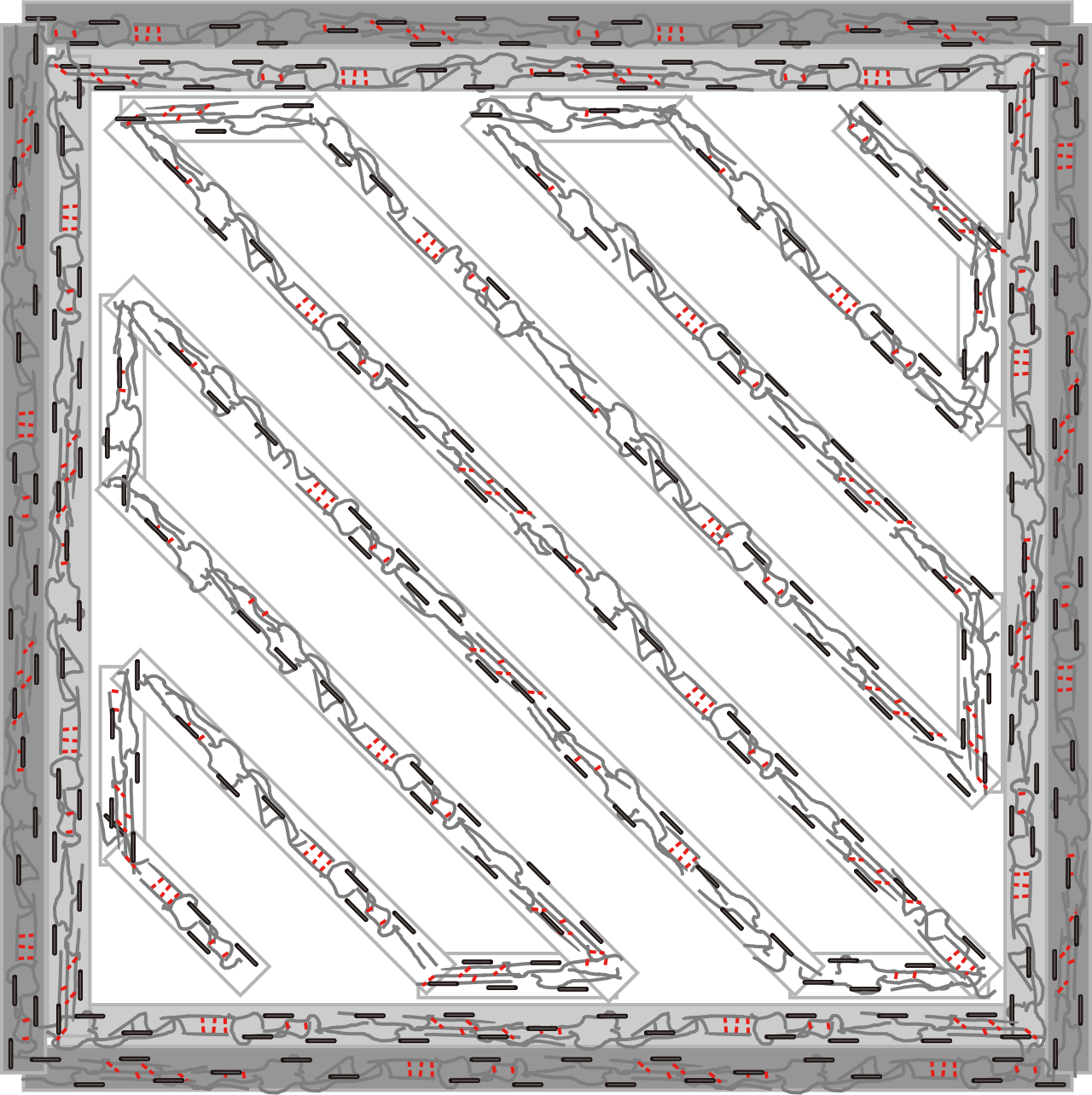** | 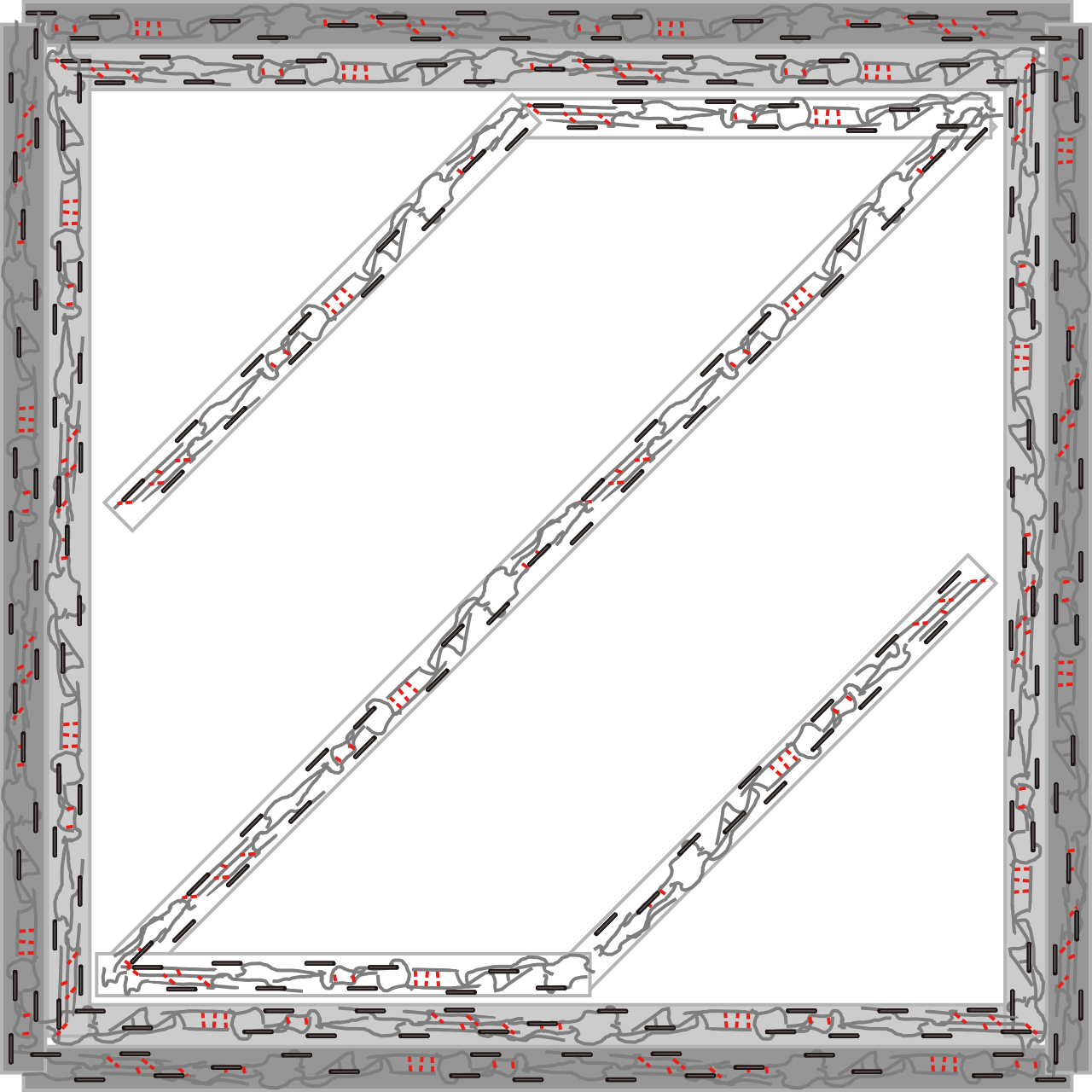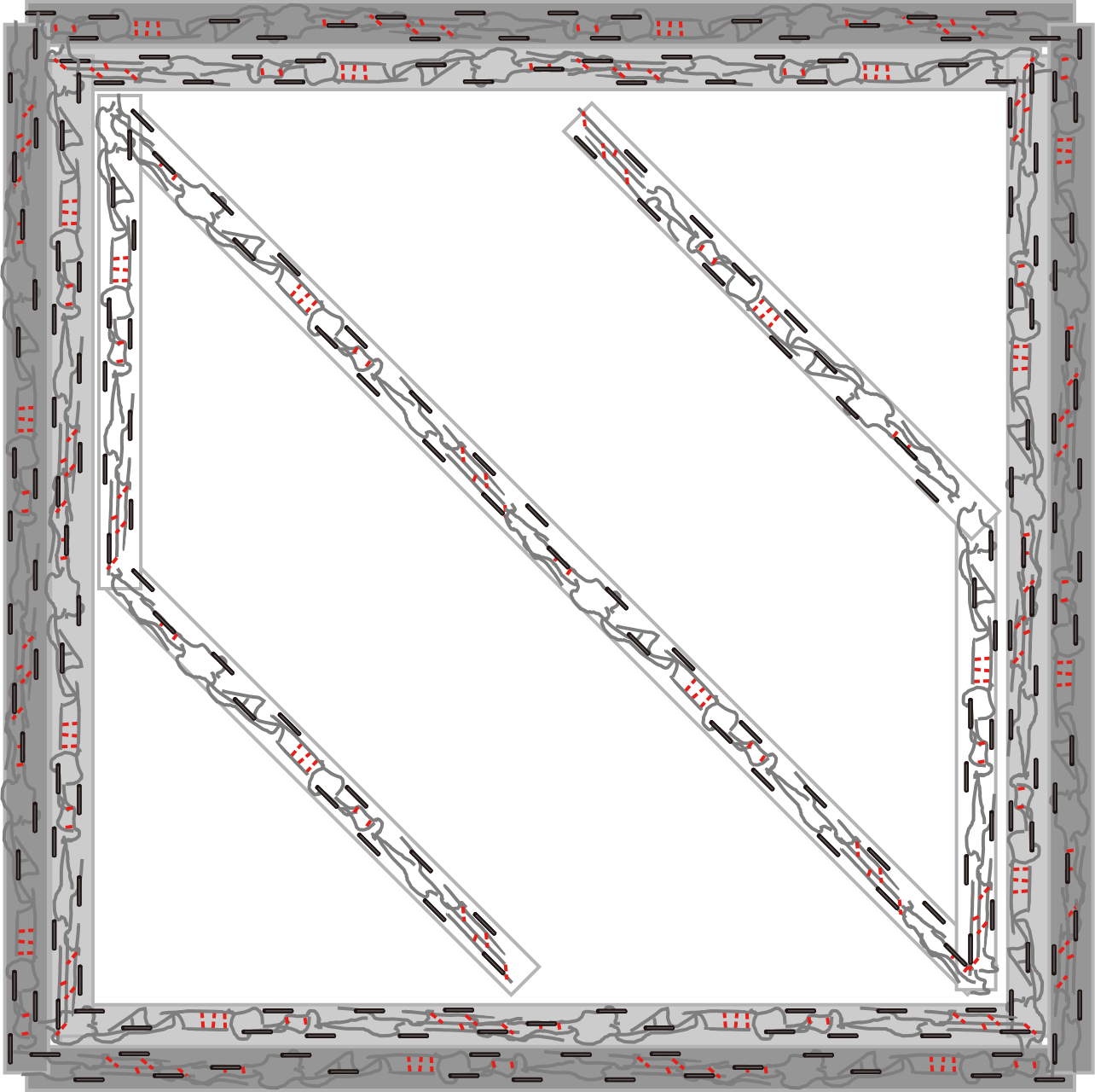 |
| **80ZG** | **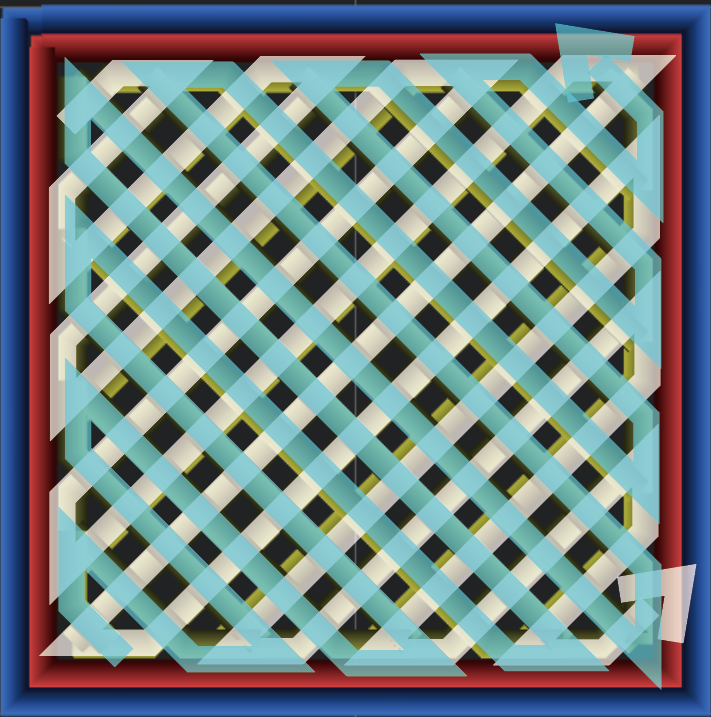** | **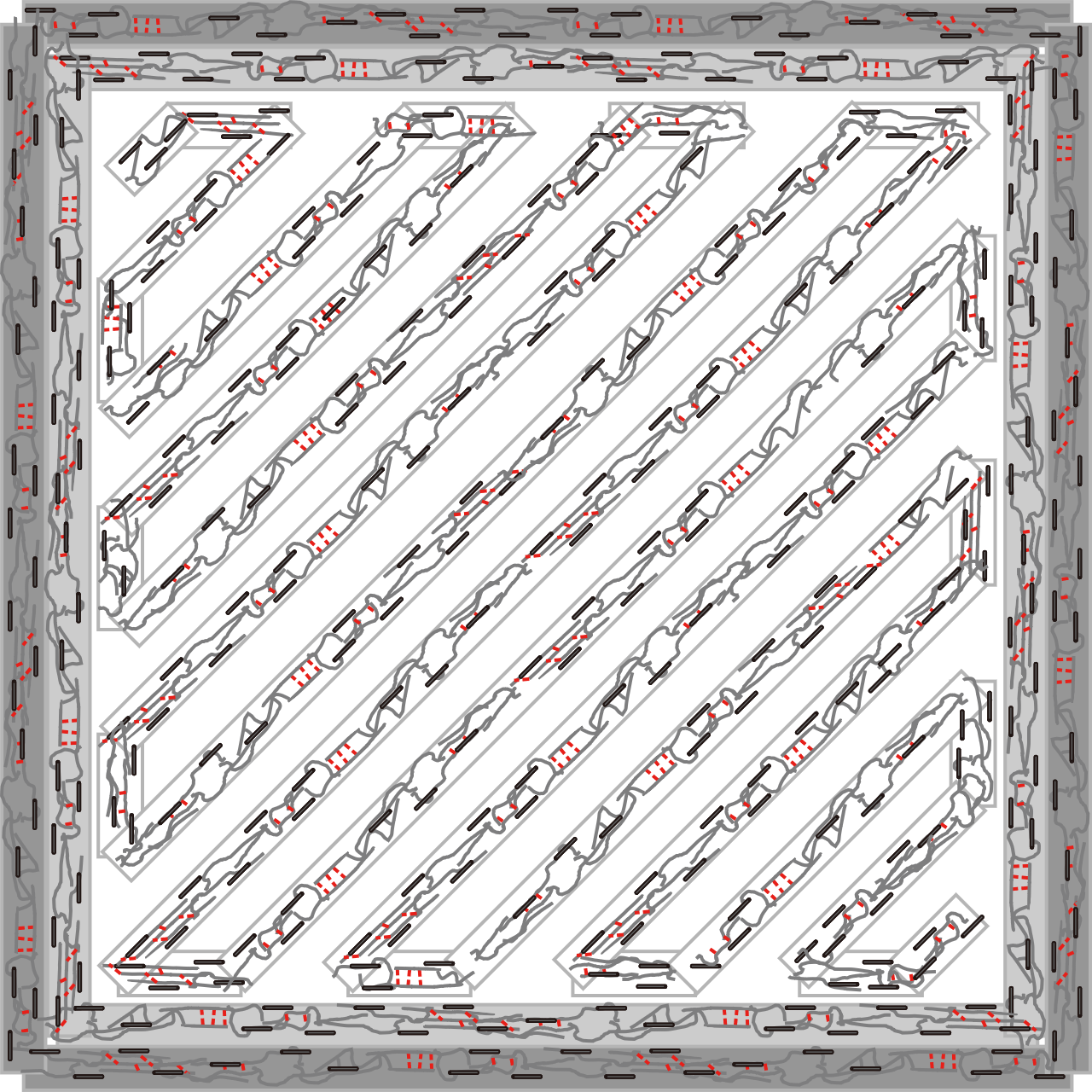** | **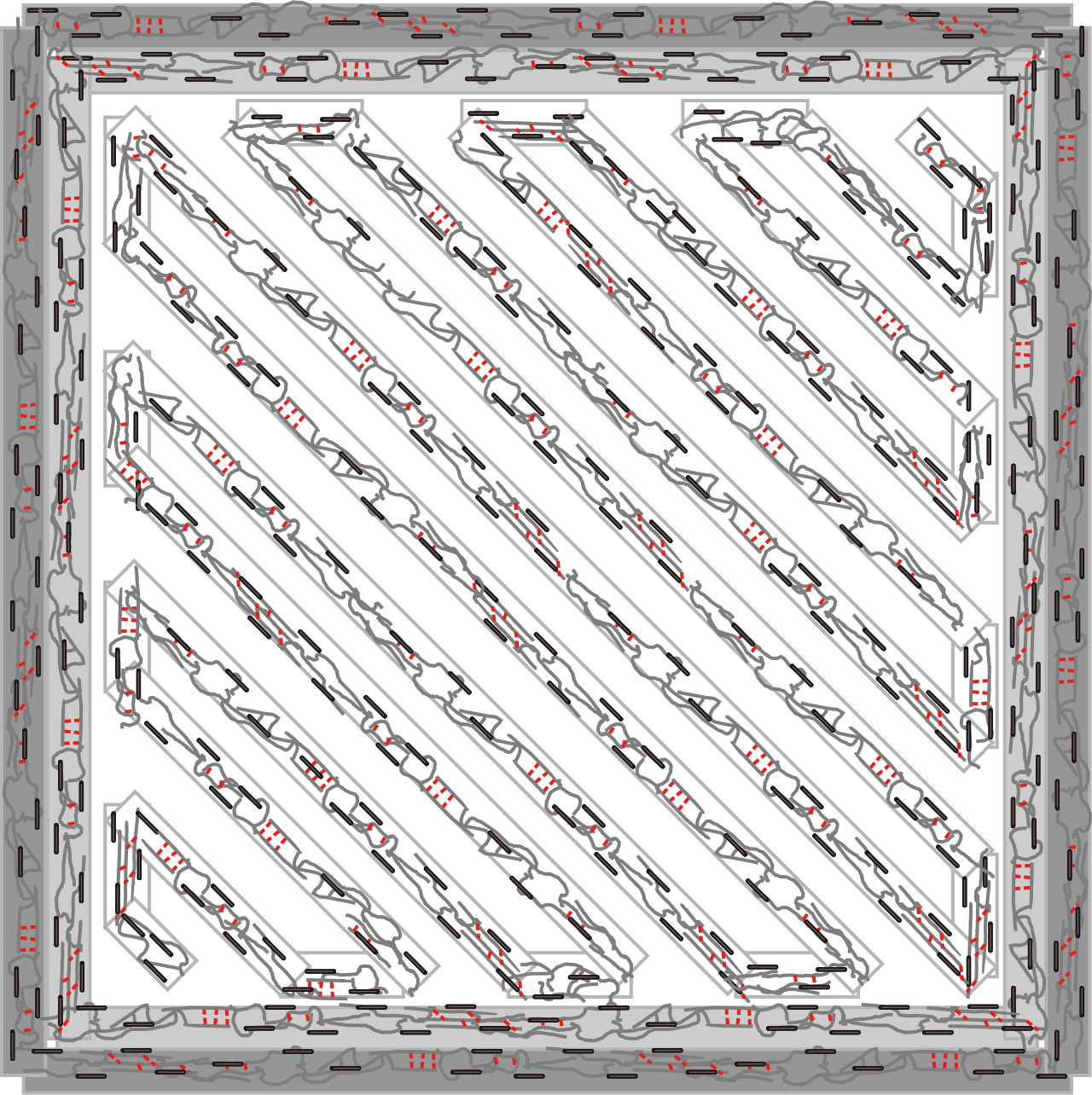** | 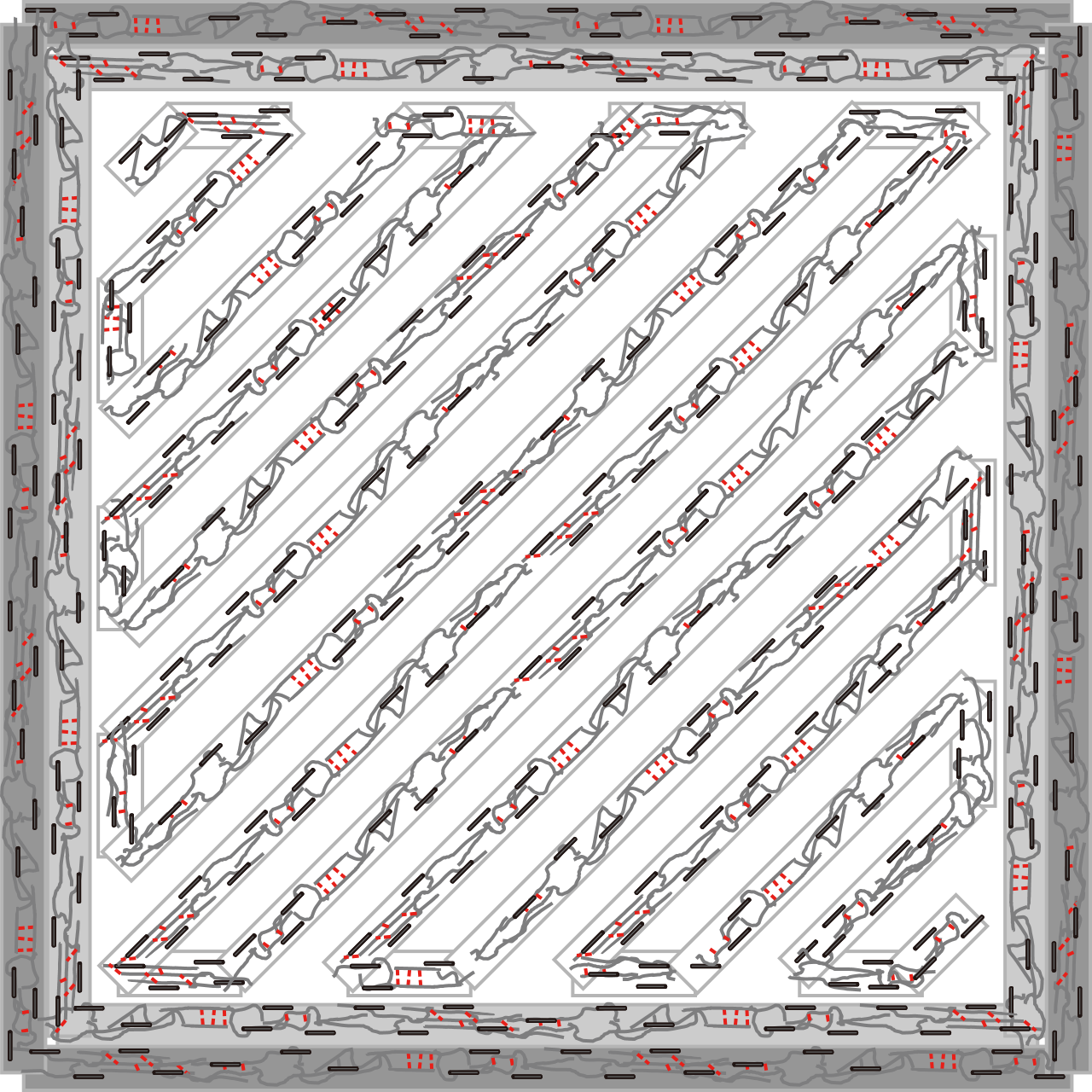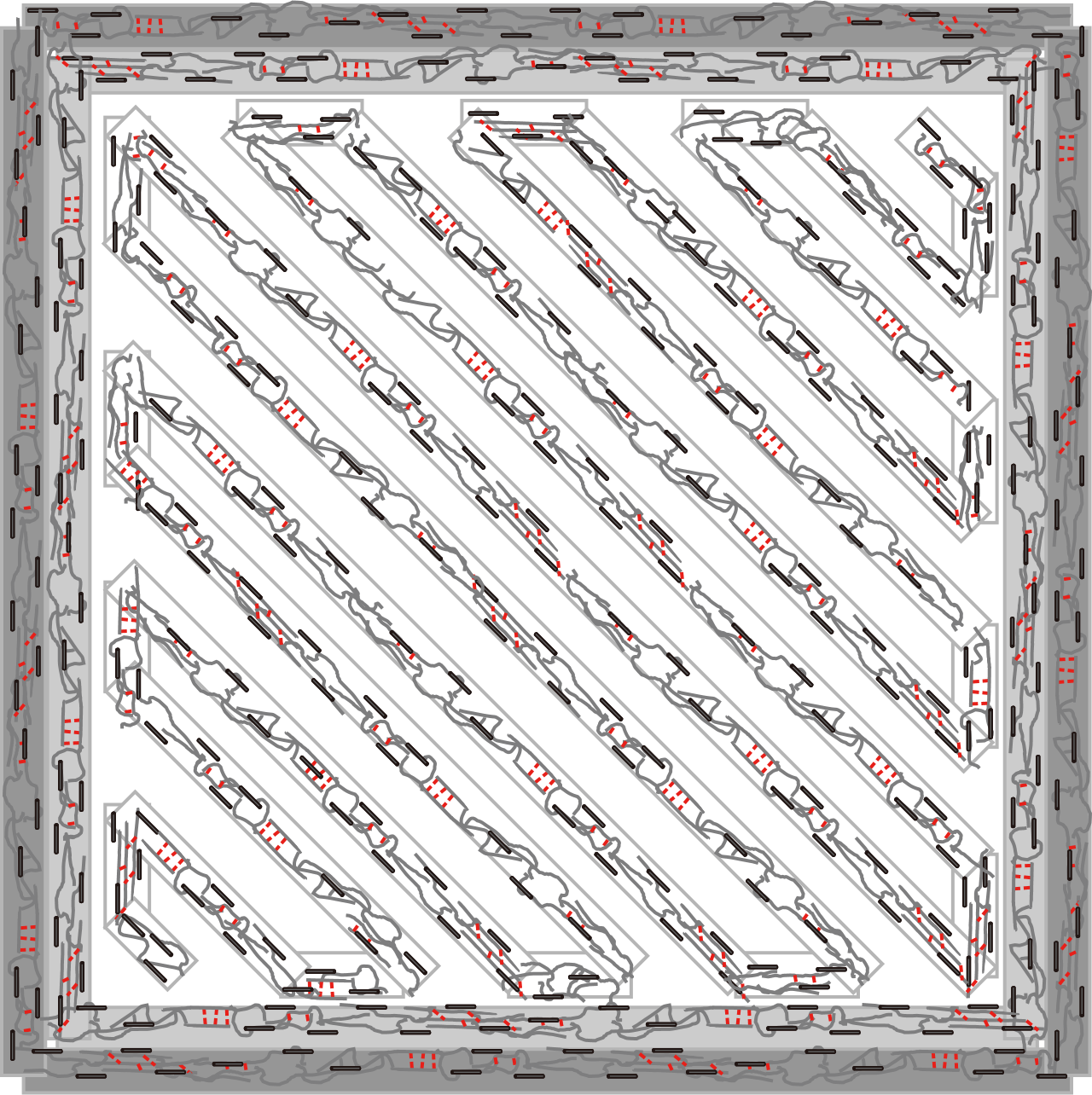 |
| **20TR** | **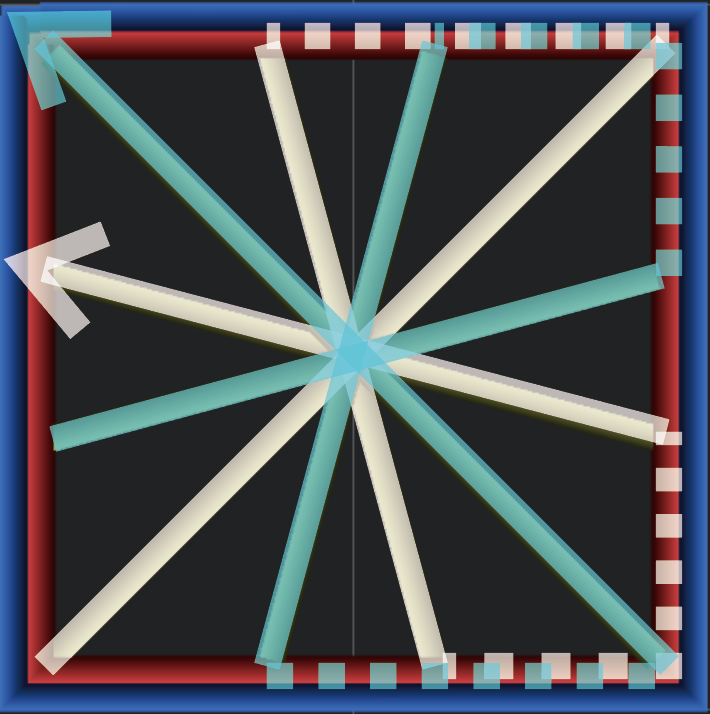** | **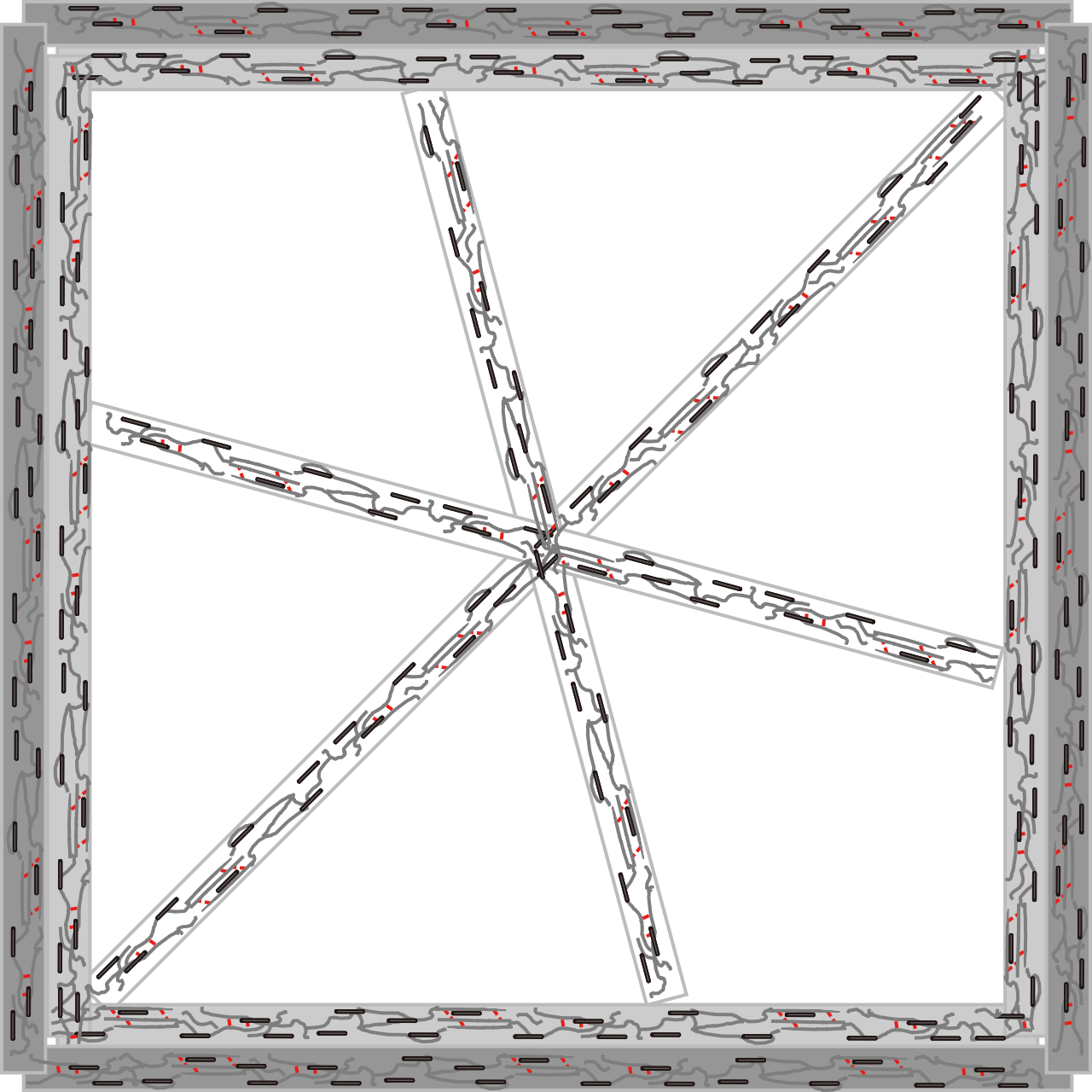** | **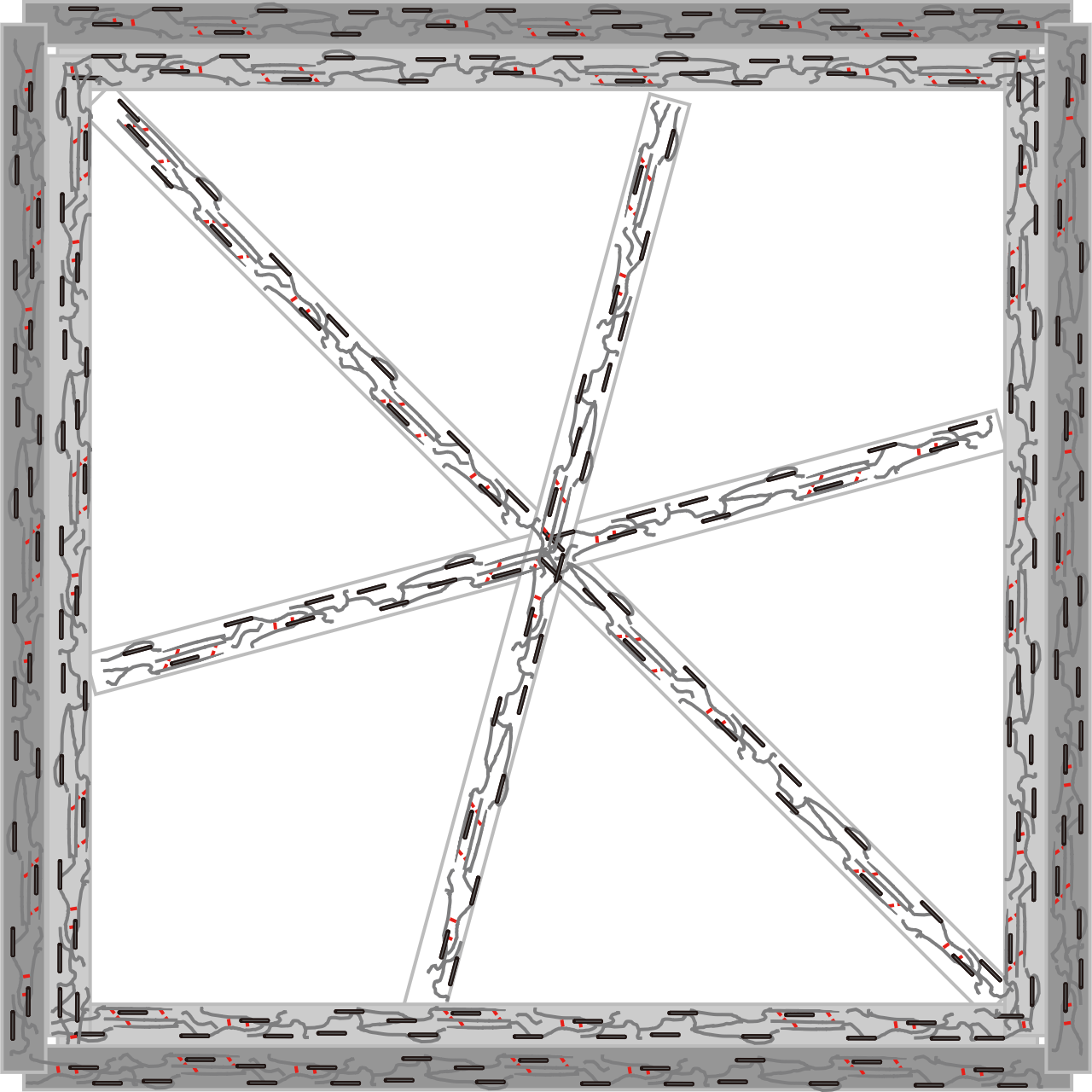** | 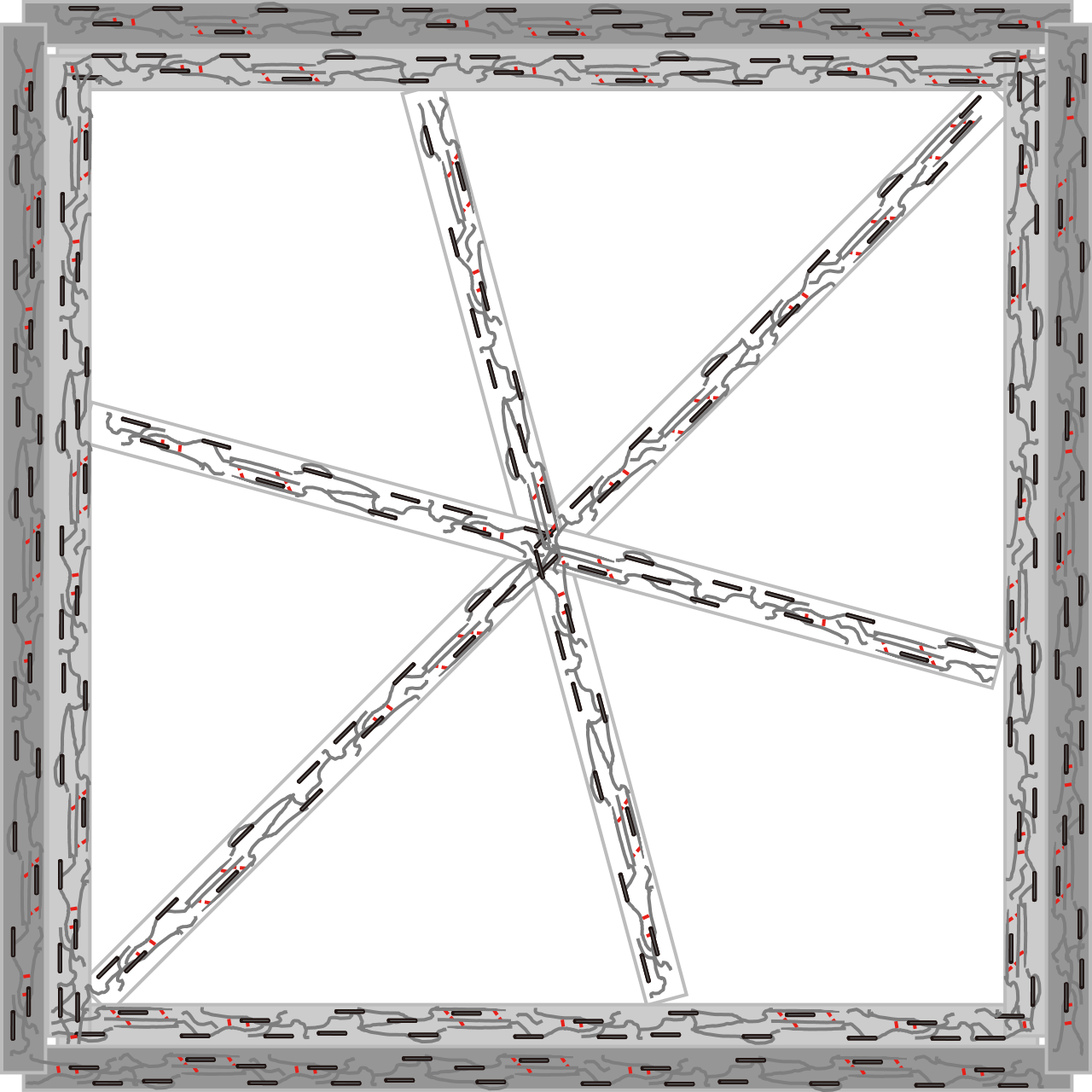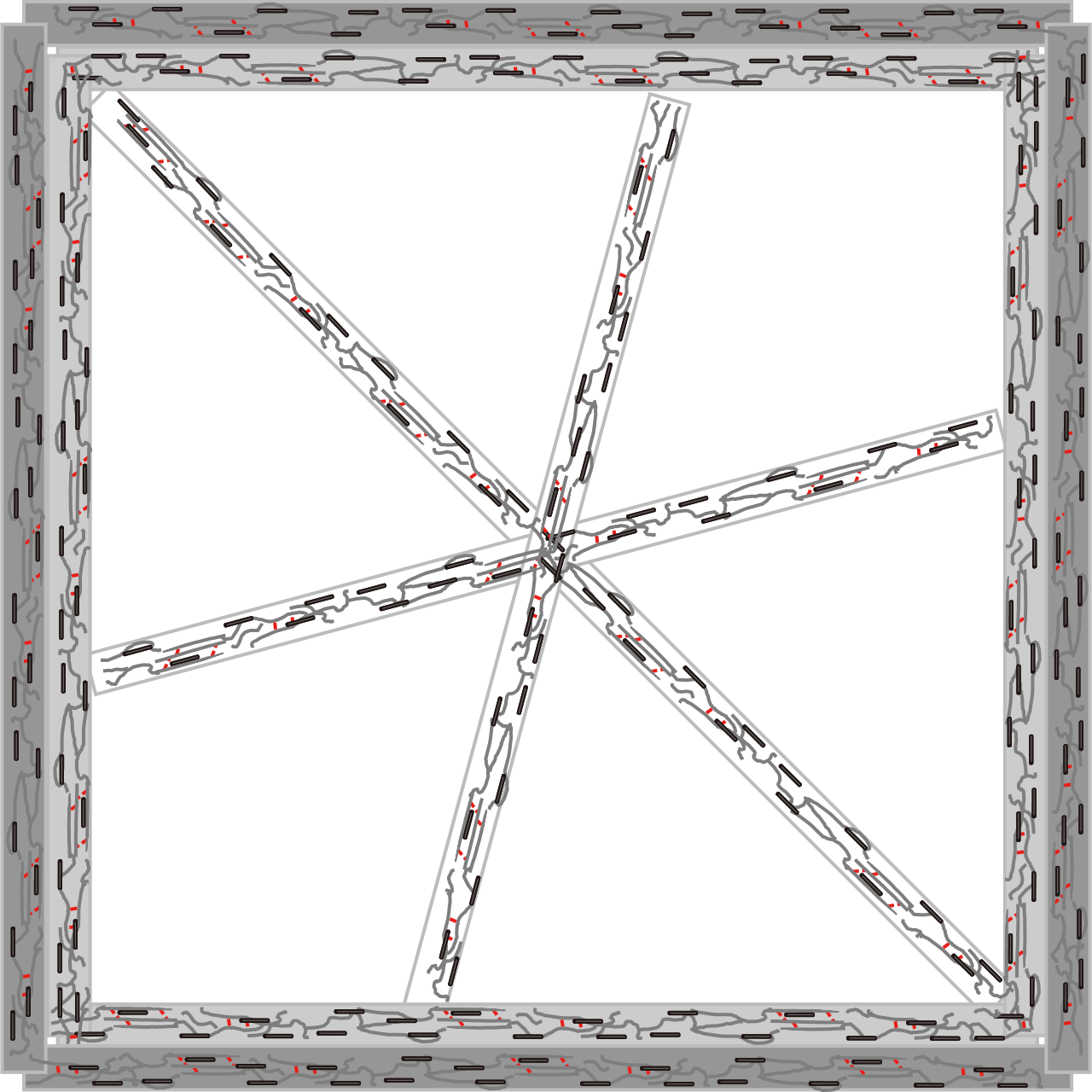 |
| **50TR** | **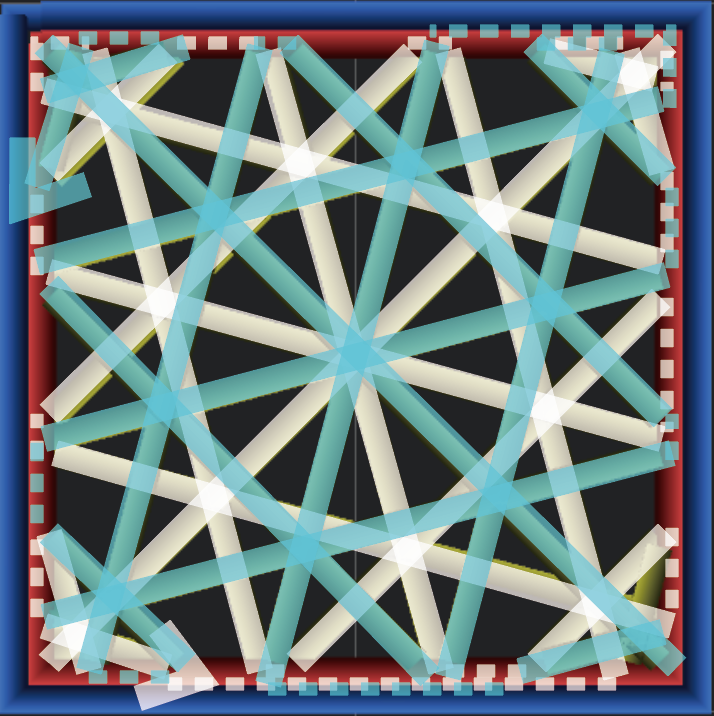** | **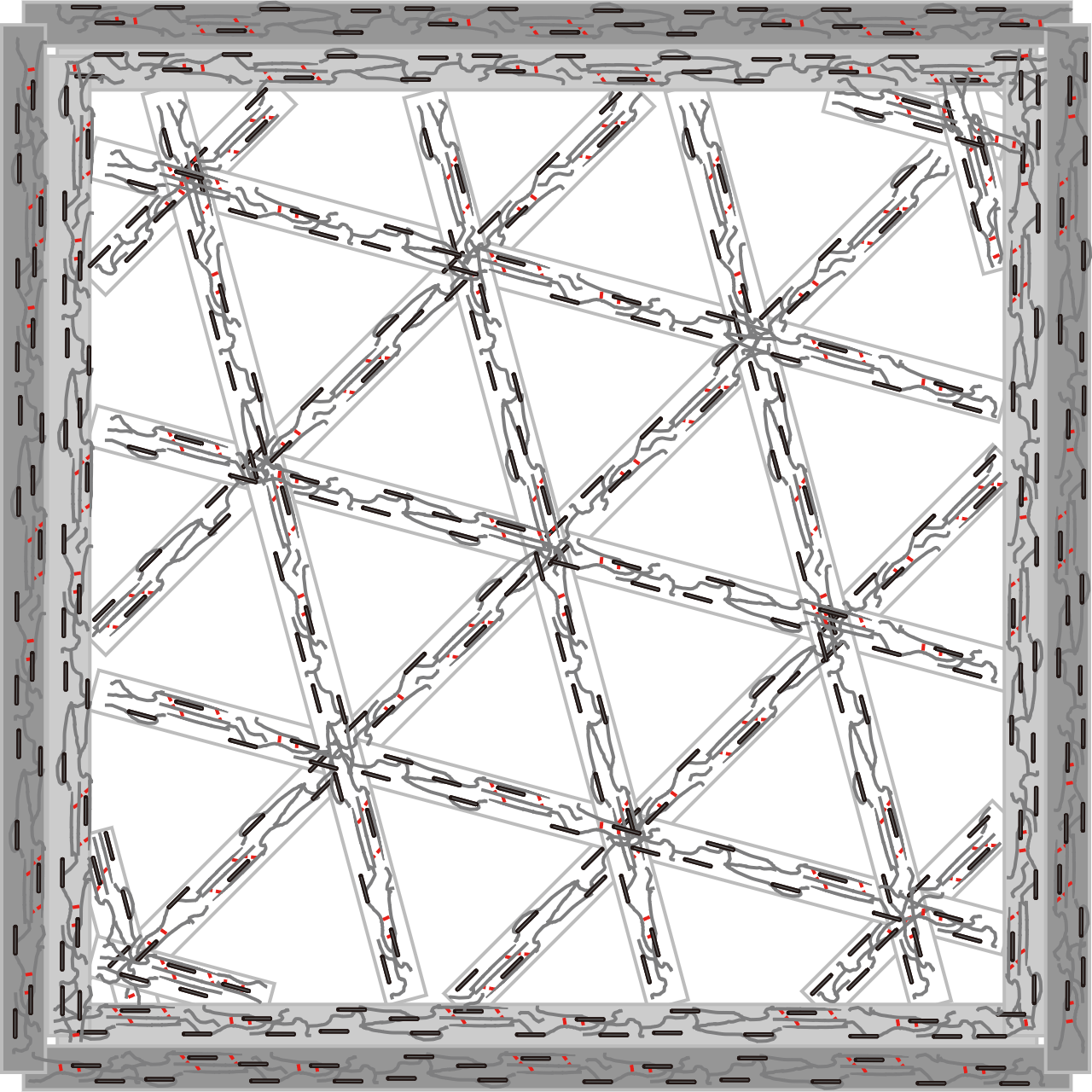** | **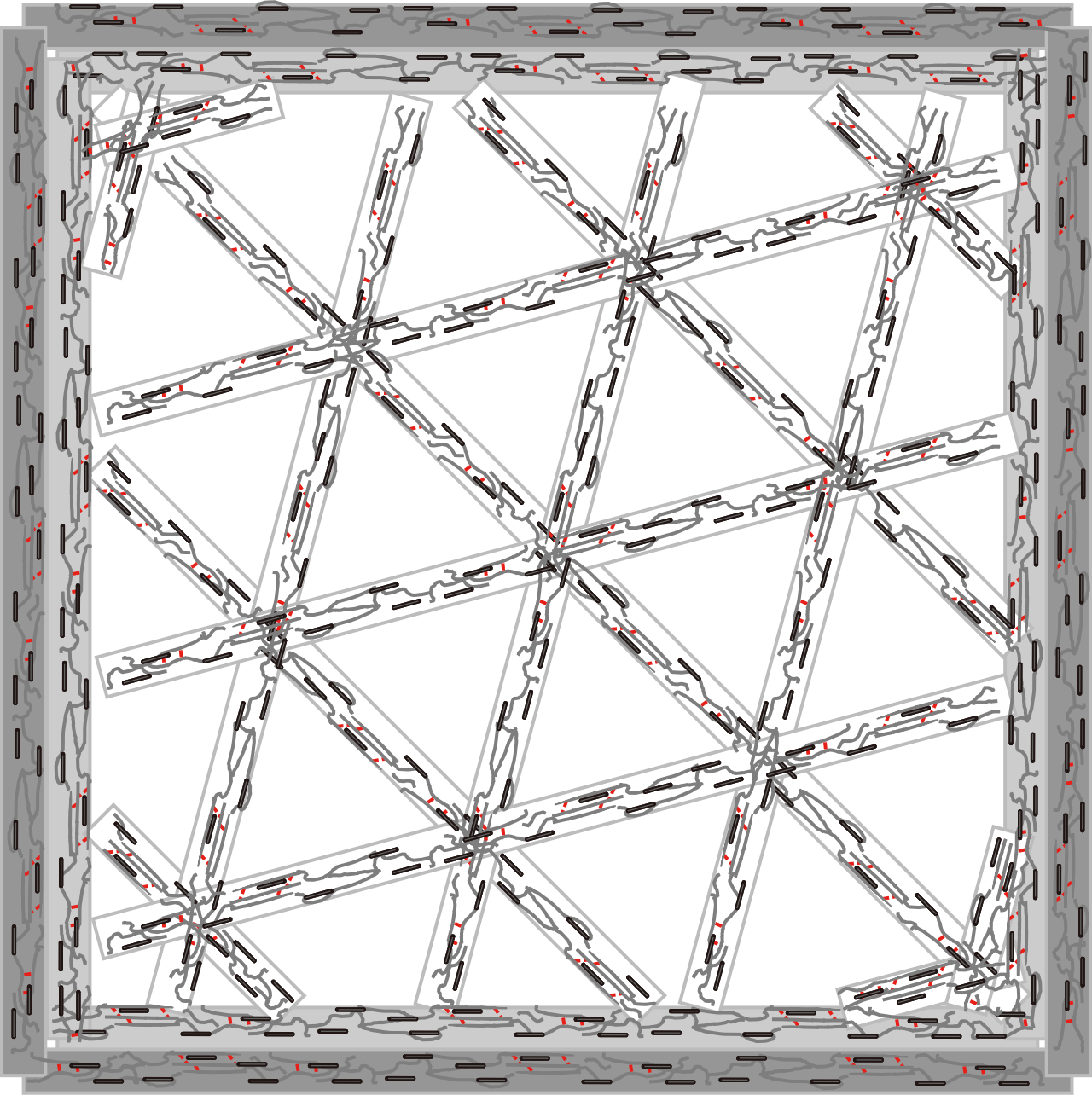** | 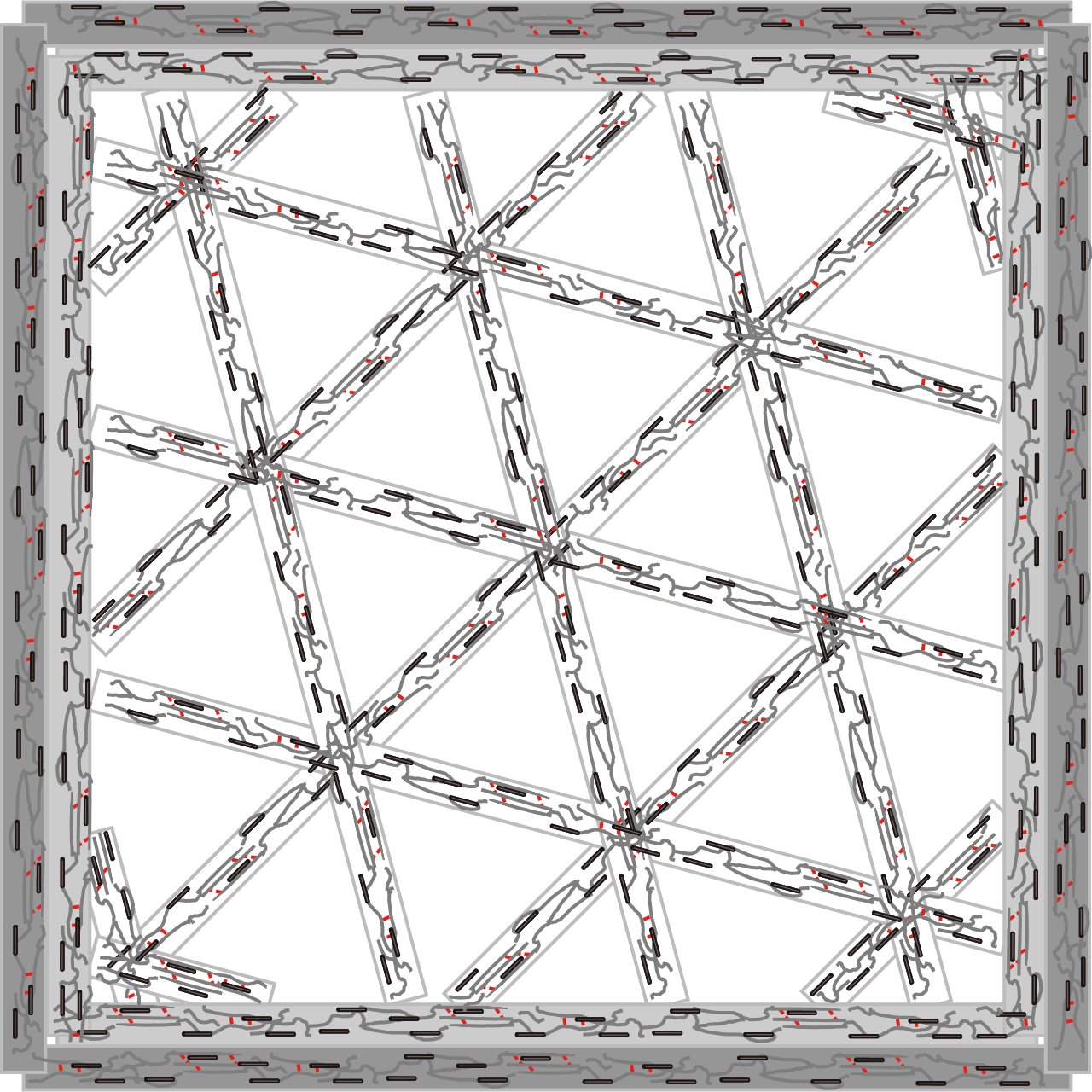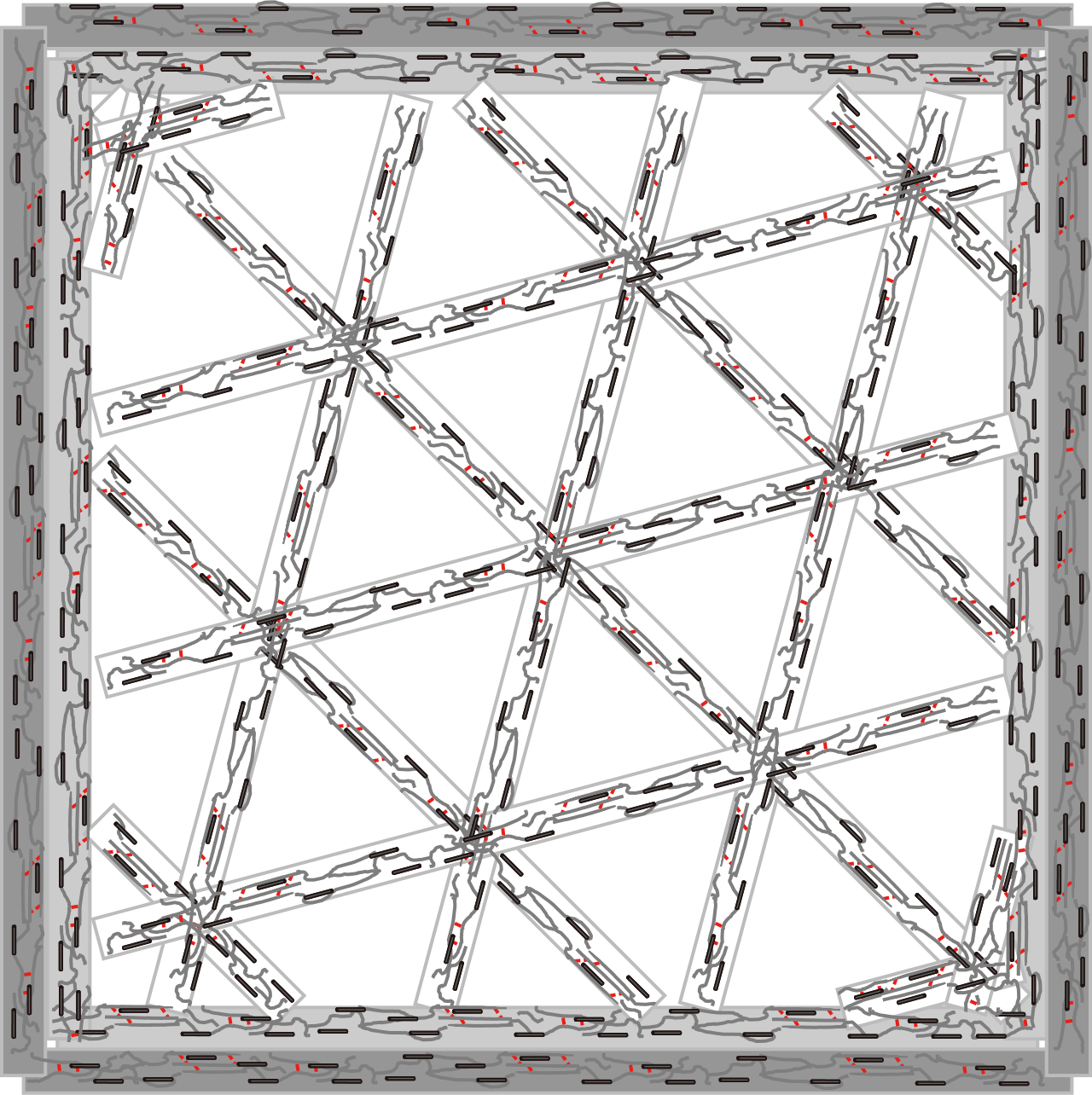 |
| **80TR** | **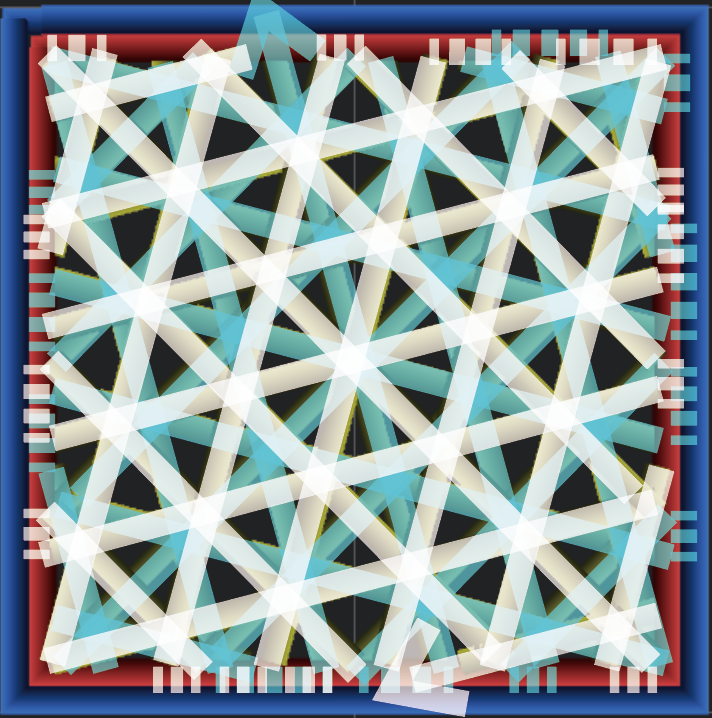** | **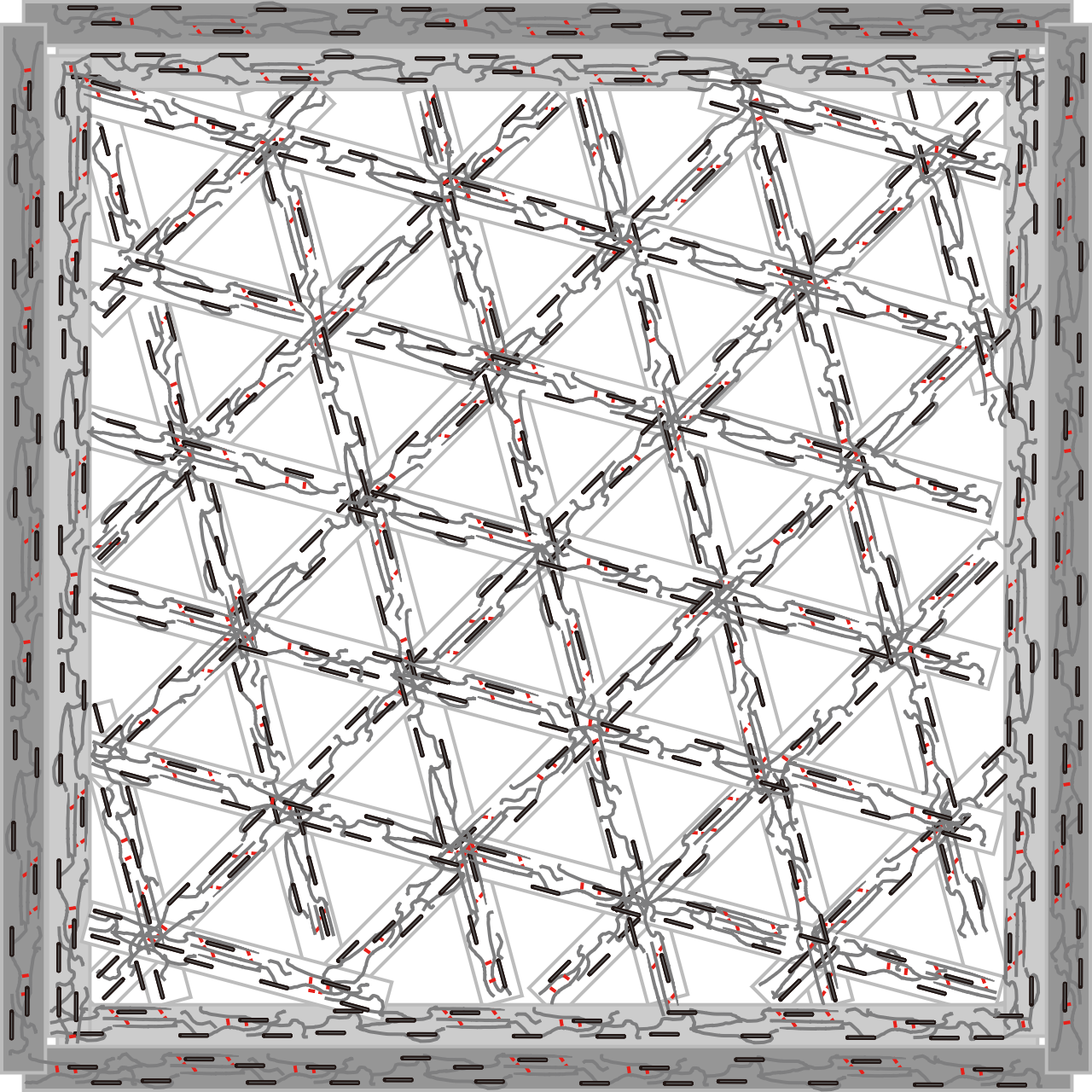** | **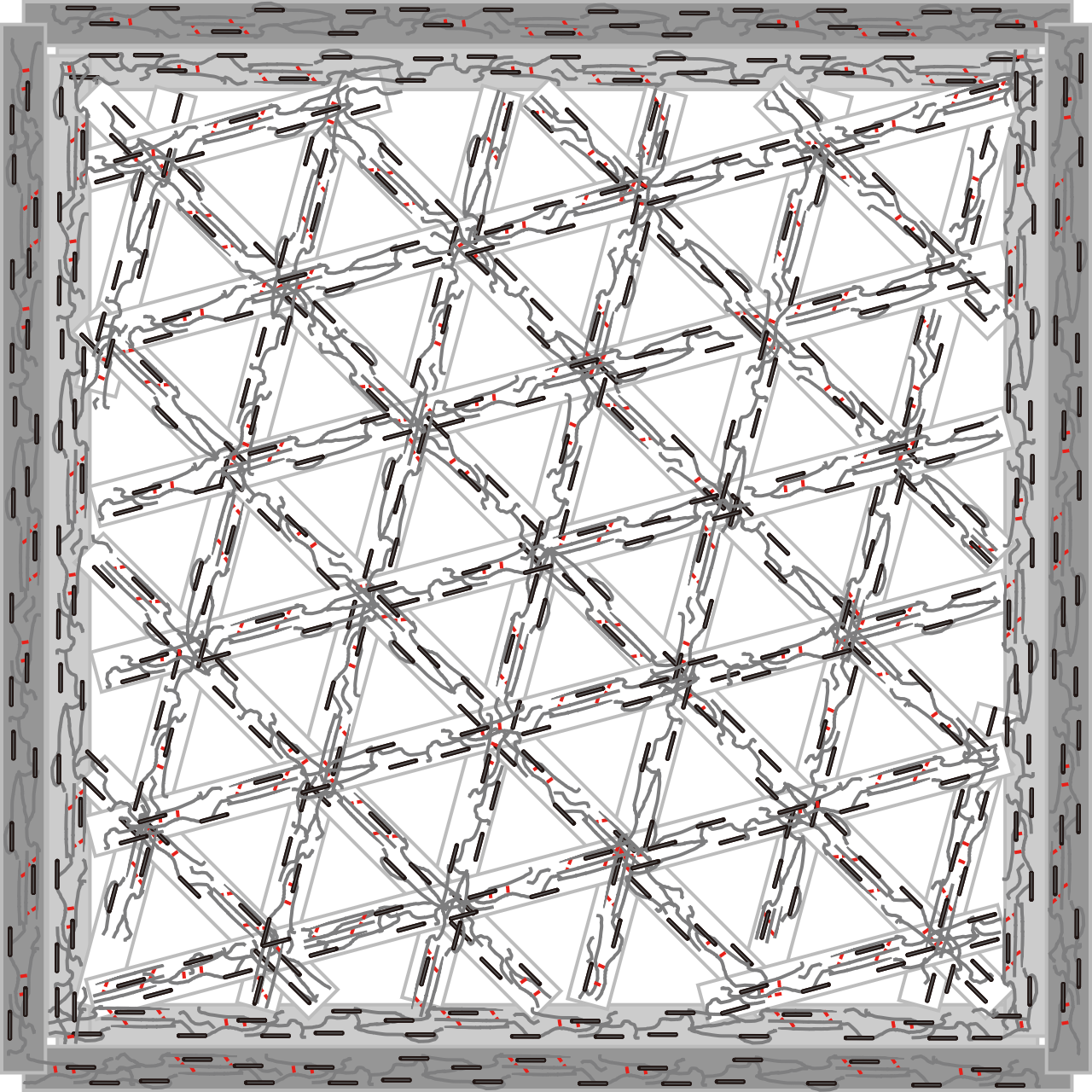** | 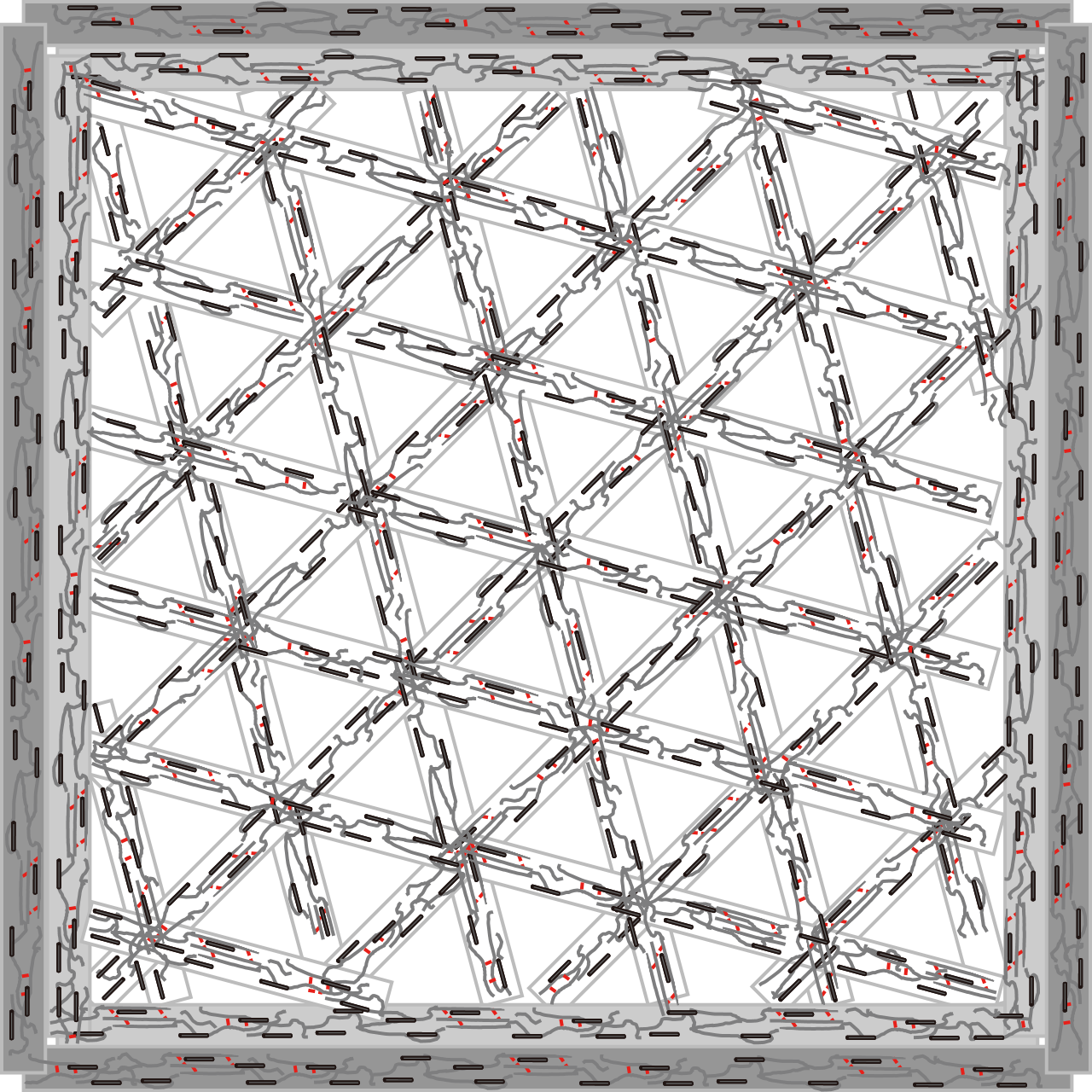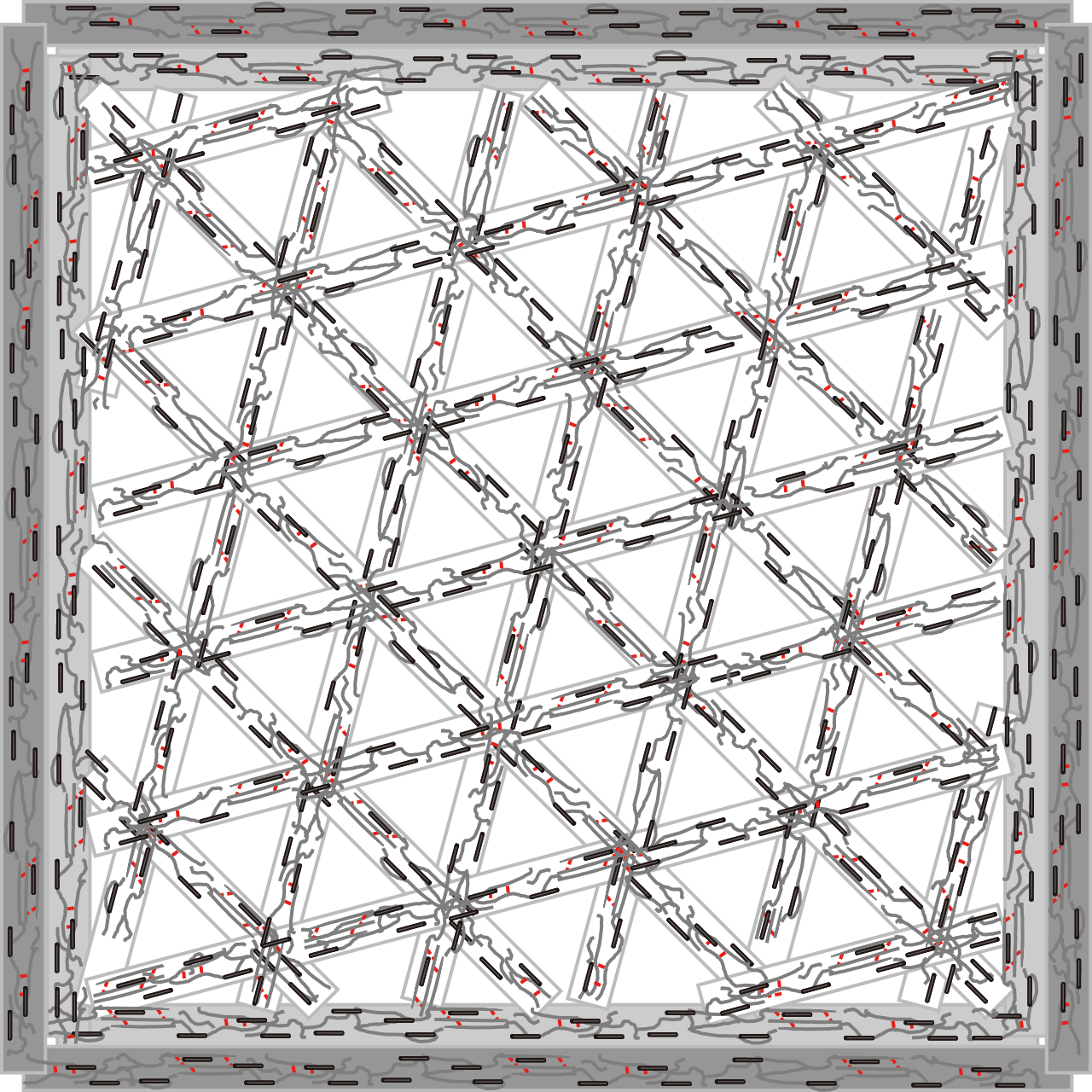 |

**Table S1**. (Continued)

| **Sample** | **Path** | **1 layer** | **2 layer** | **1+2 layer** |
| --- | --- | --- | --- | --- |
| **20HN** | **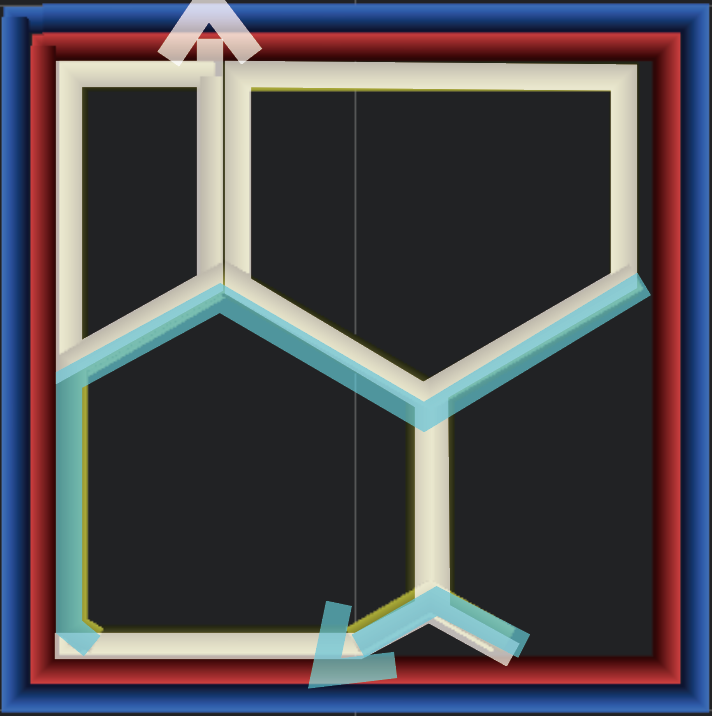** | **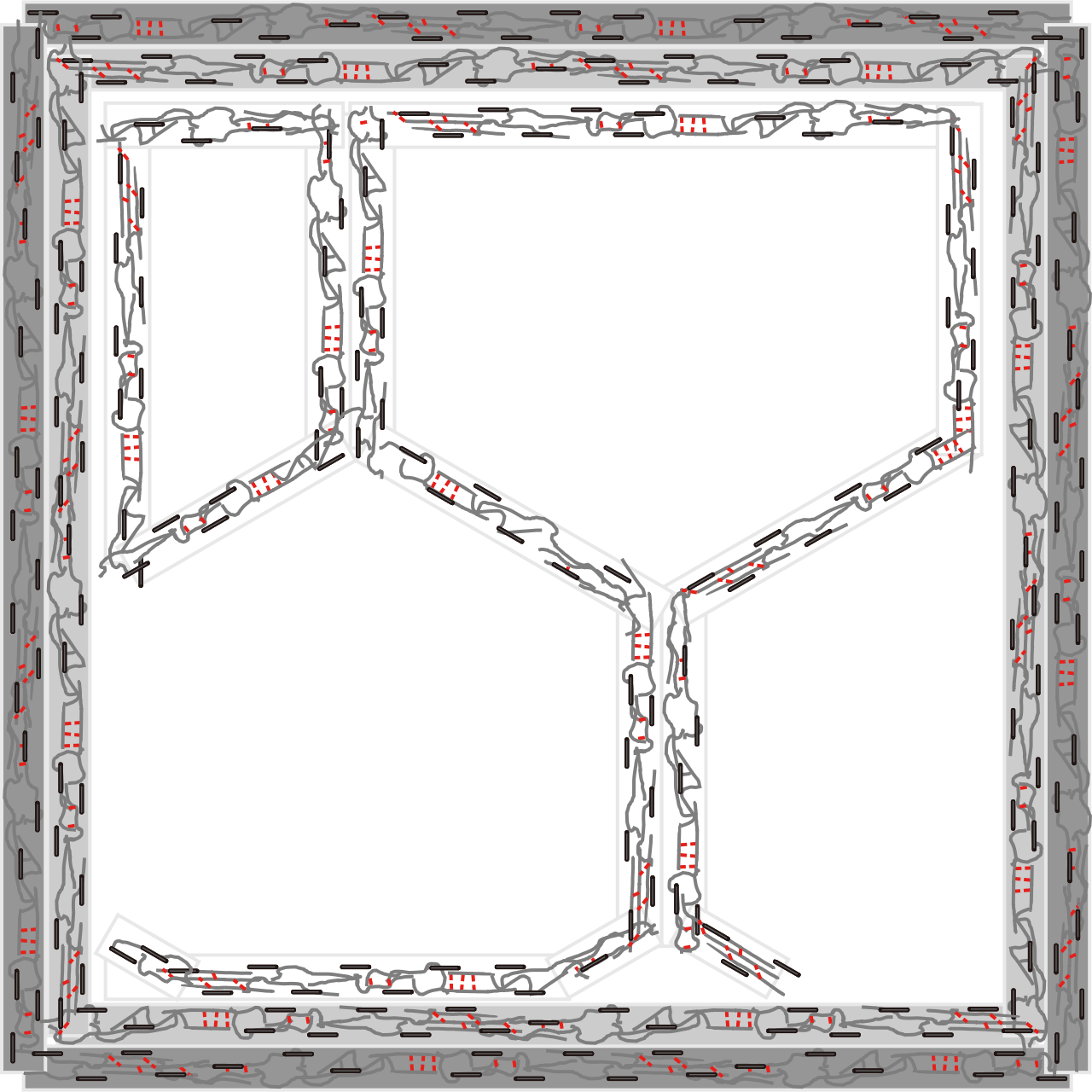** | **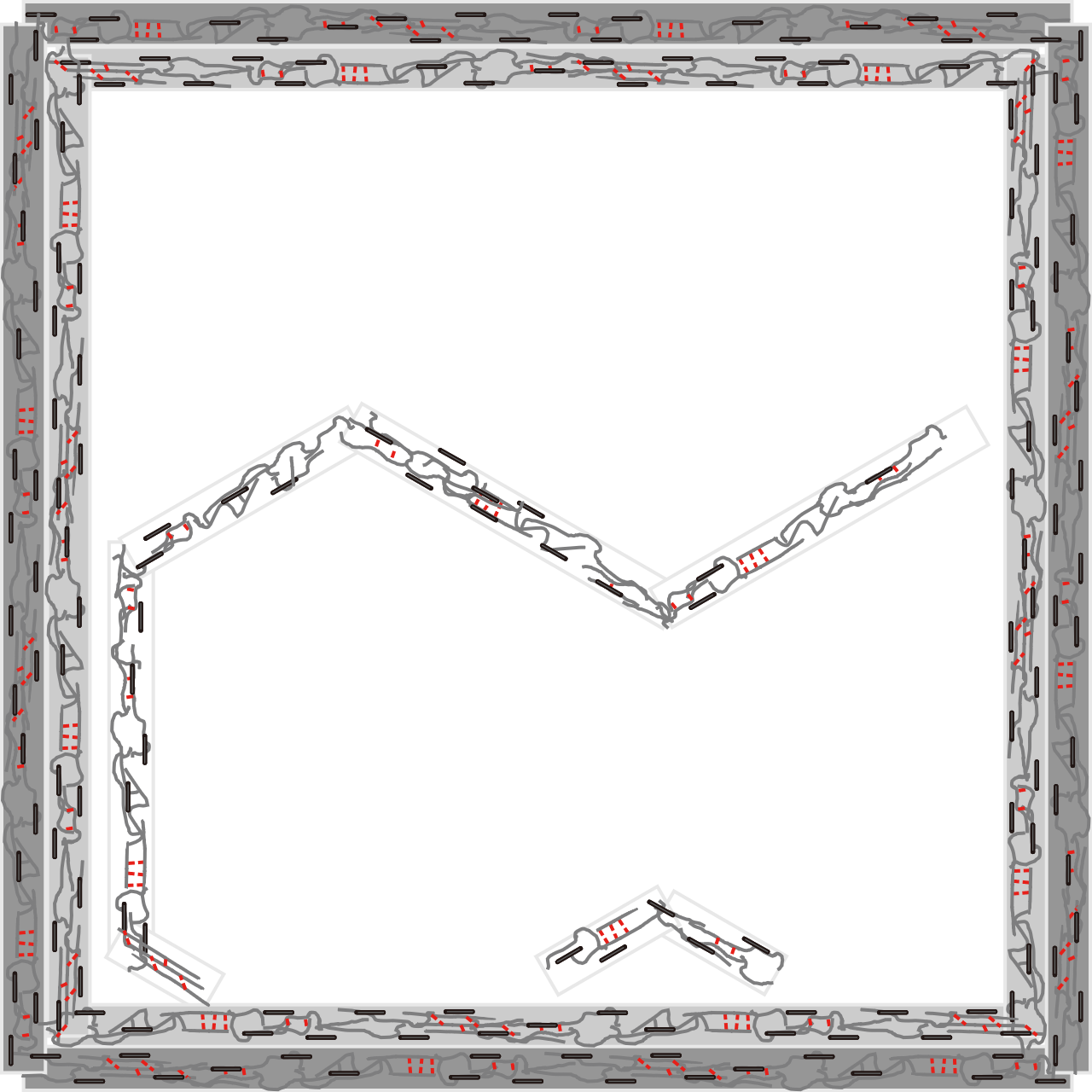** | 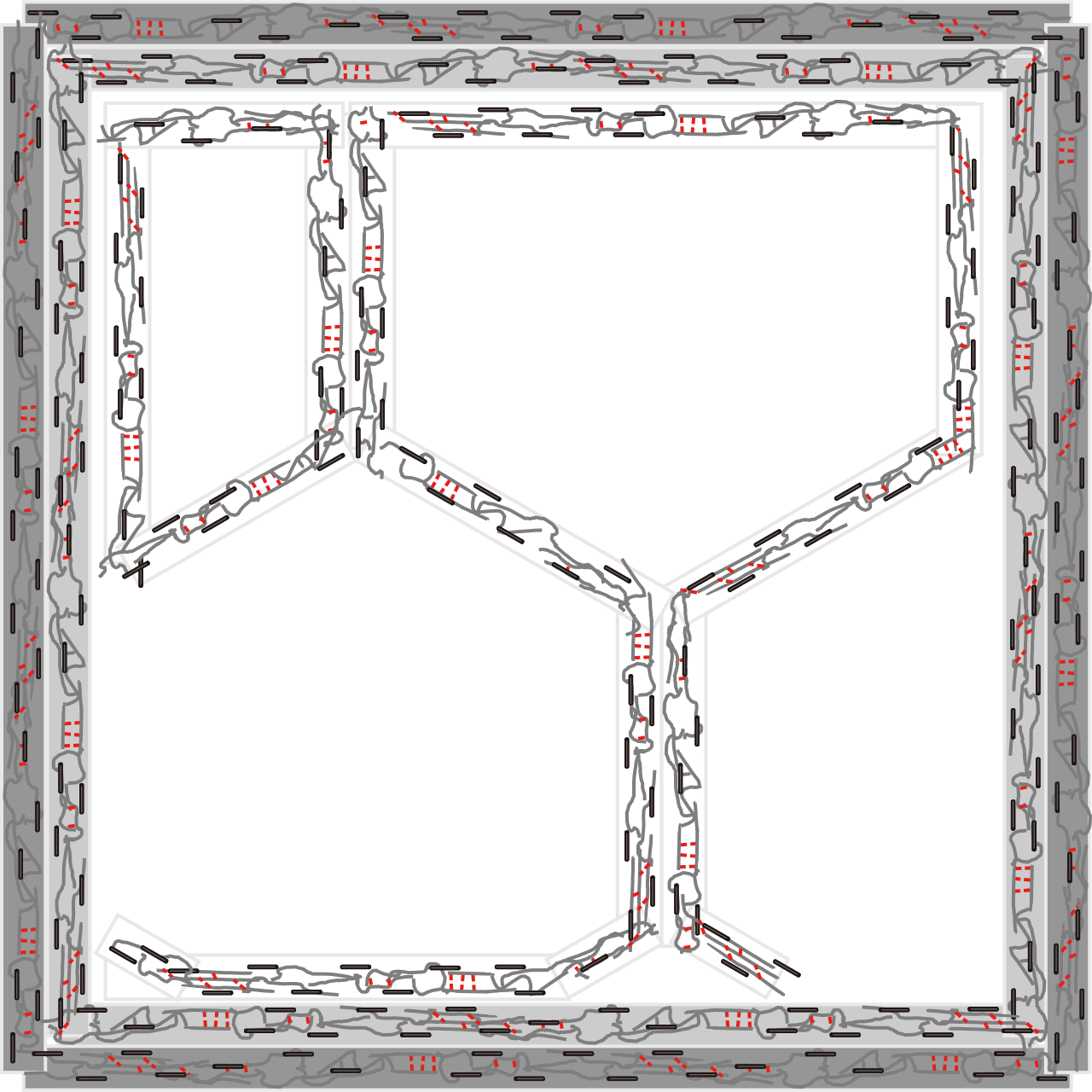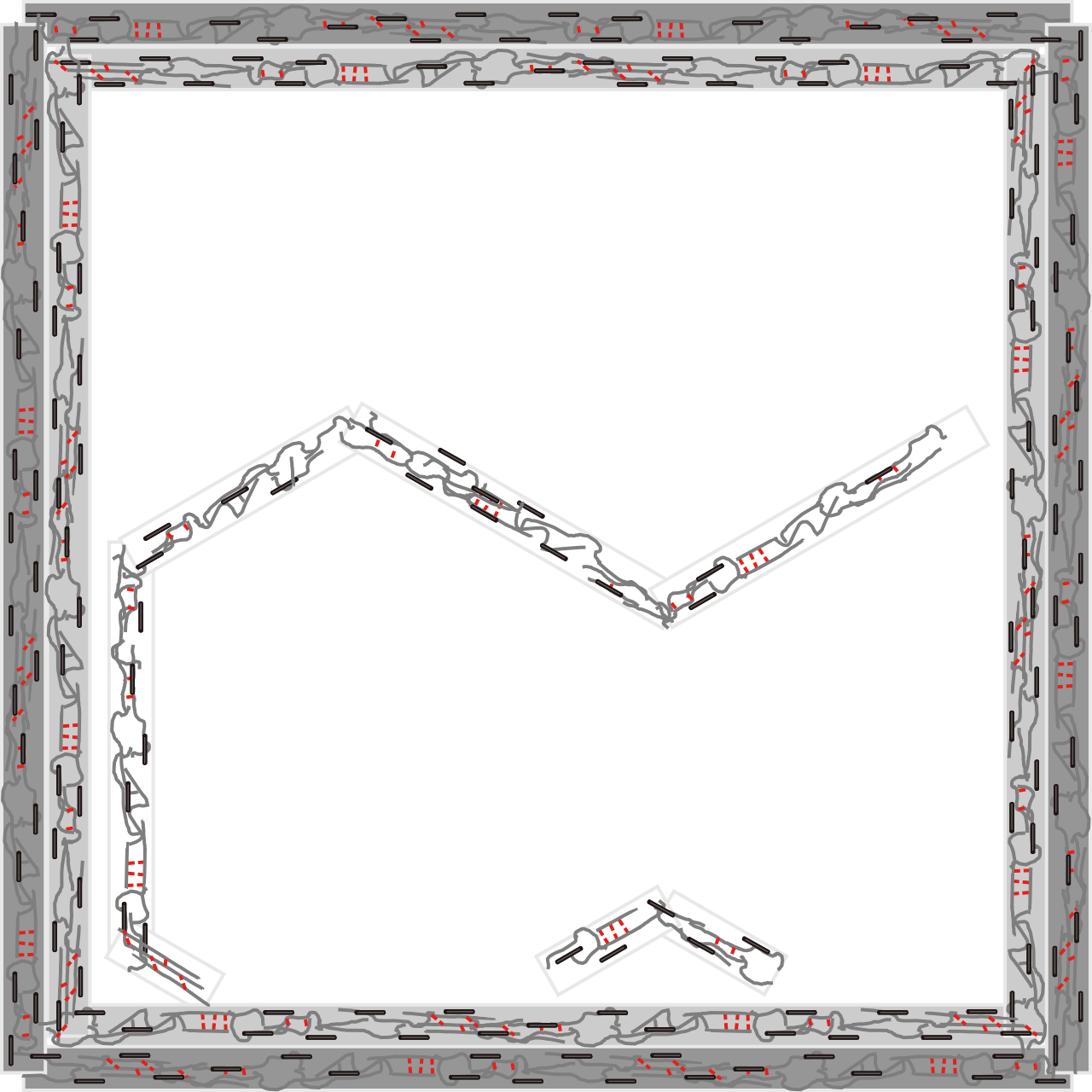 |
| **50HN** | **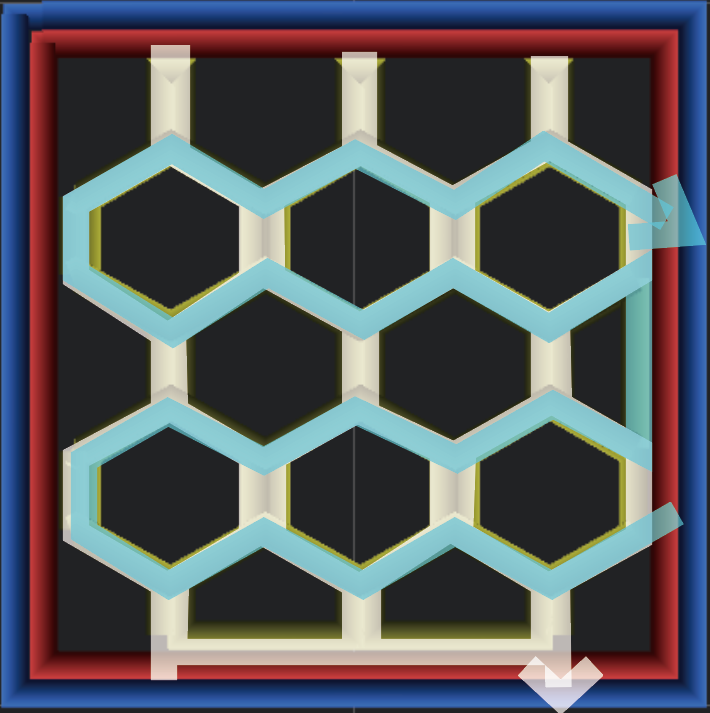** | **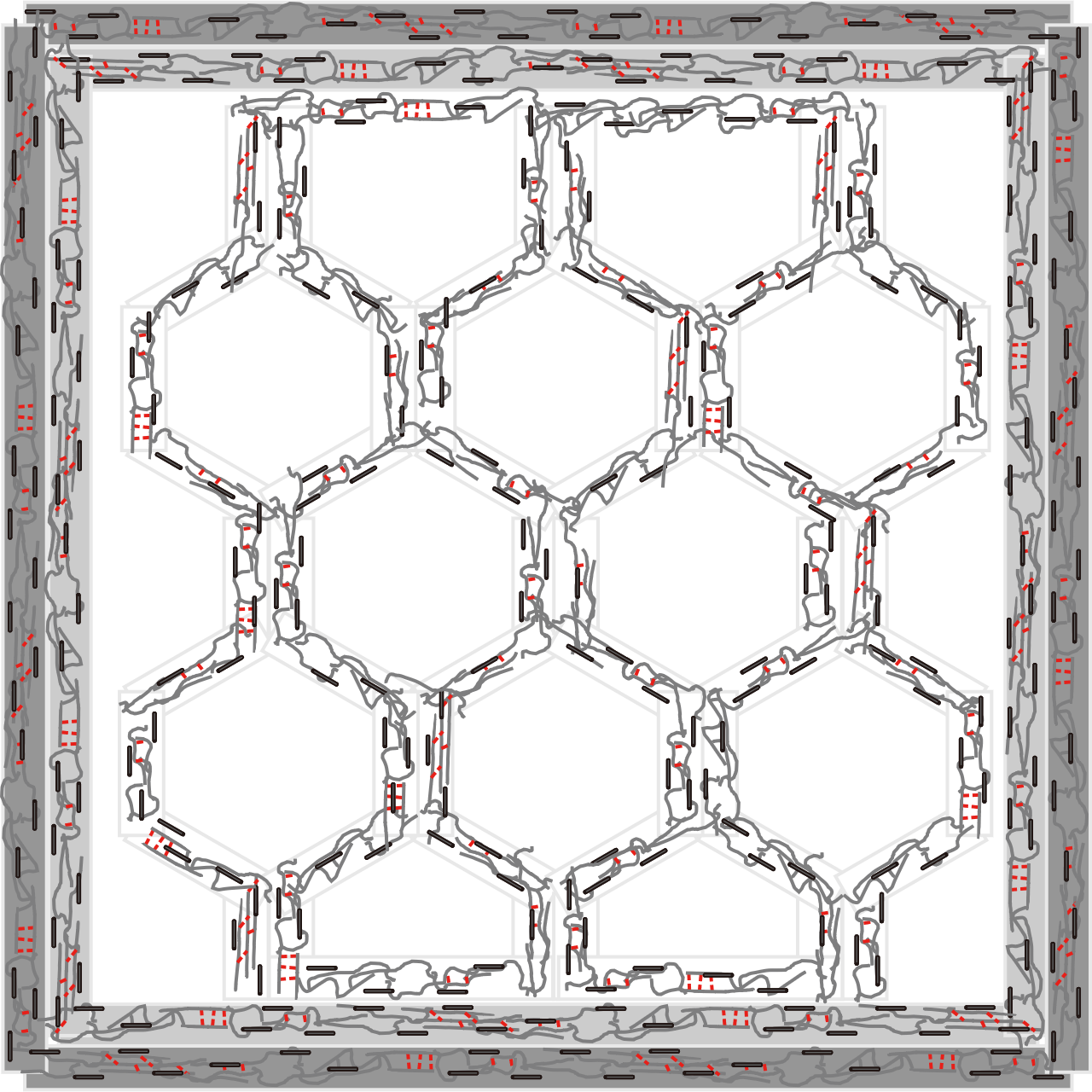** | **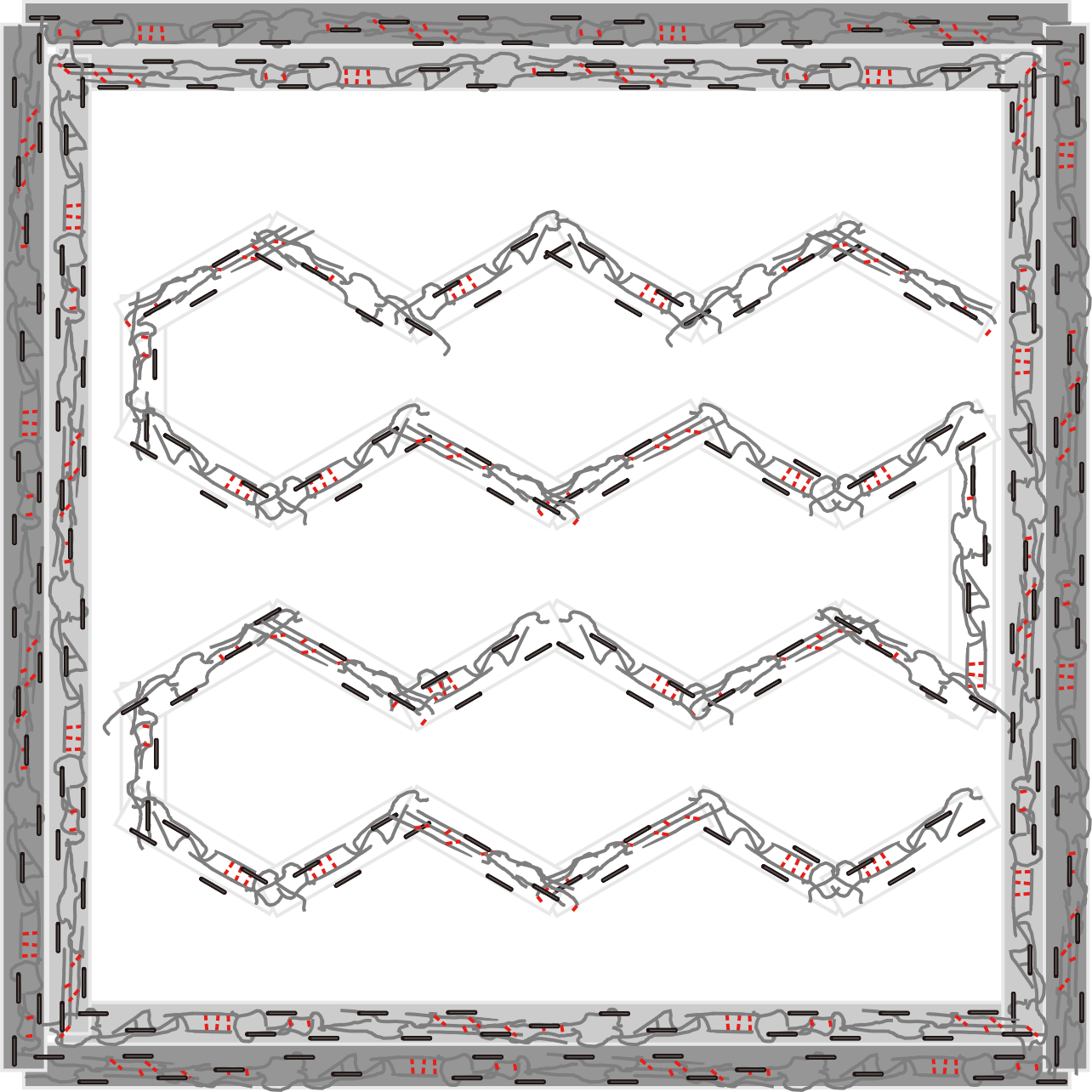** | 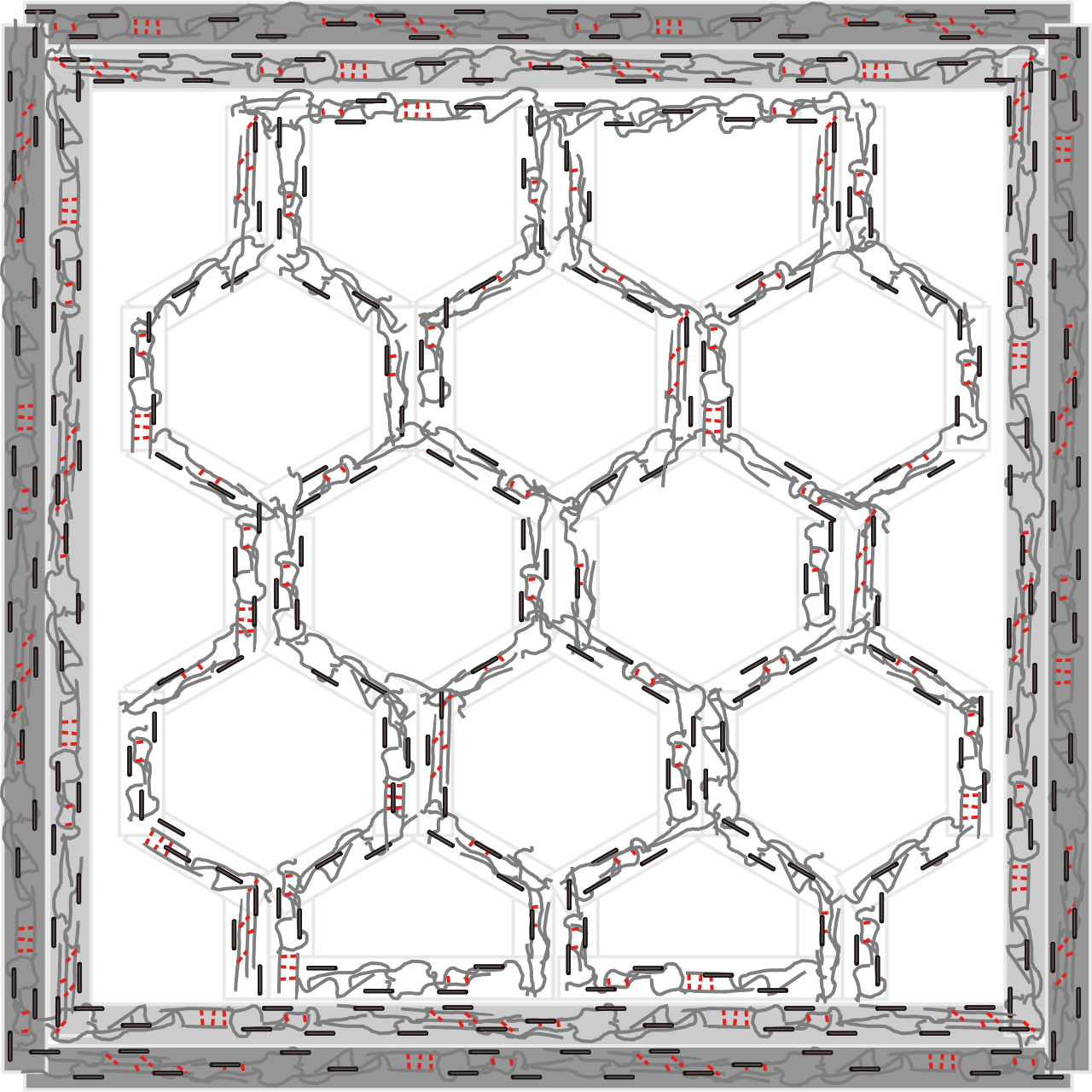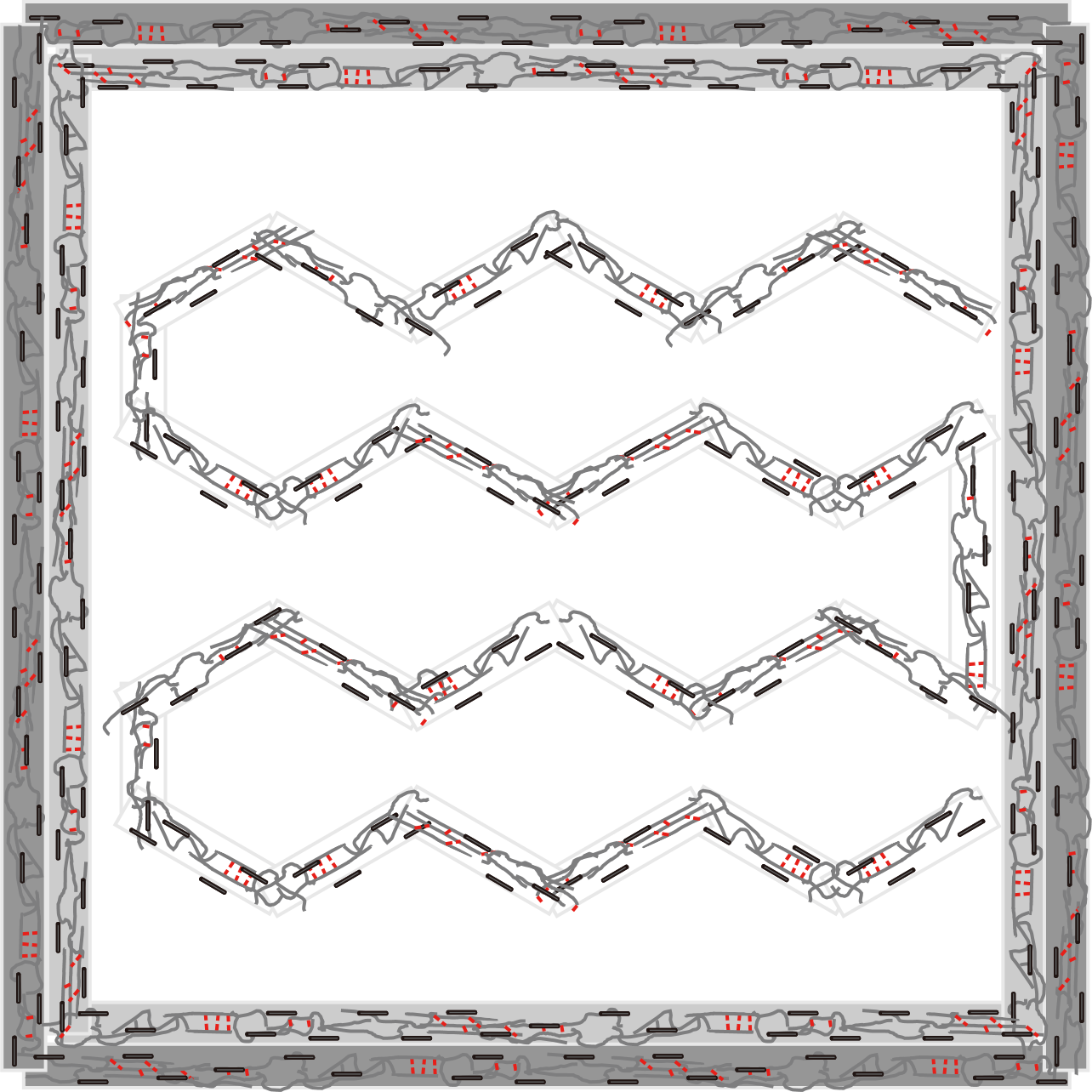 |
| **80HN** | **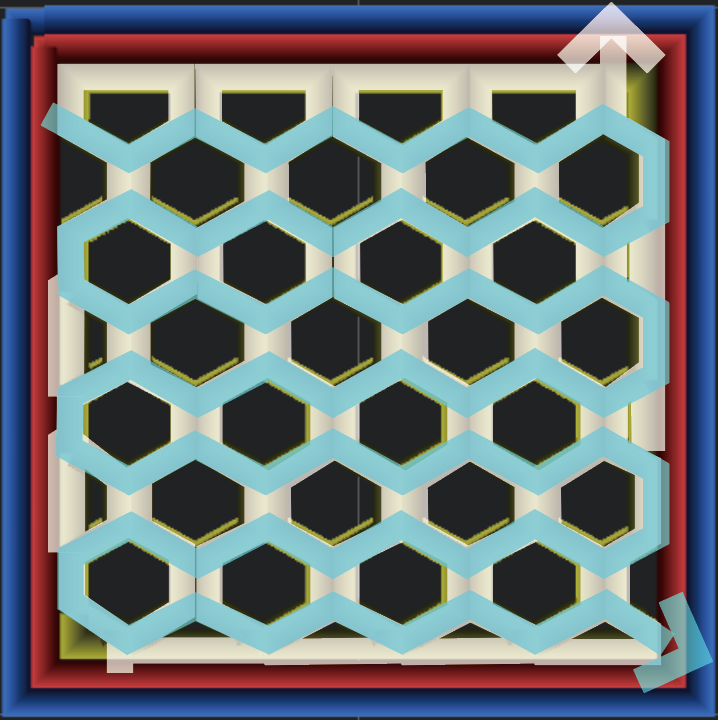** | **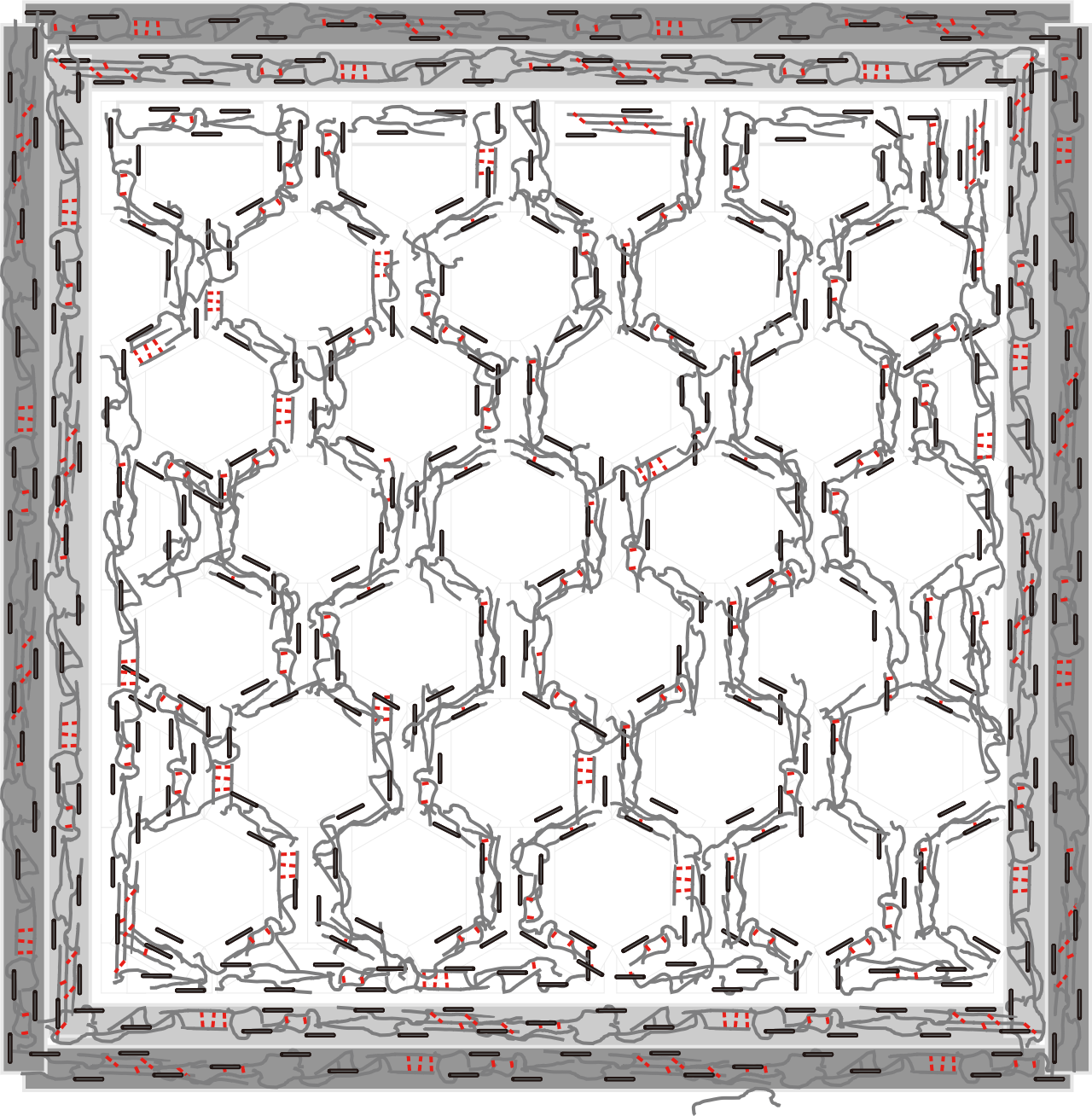** | **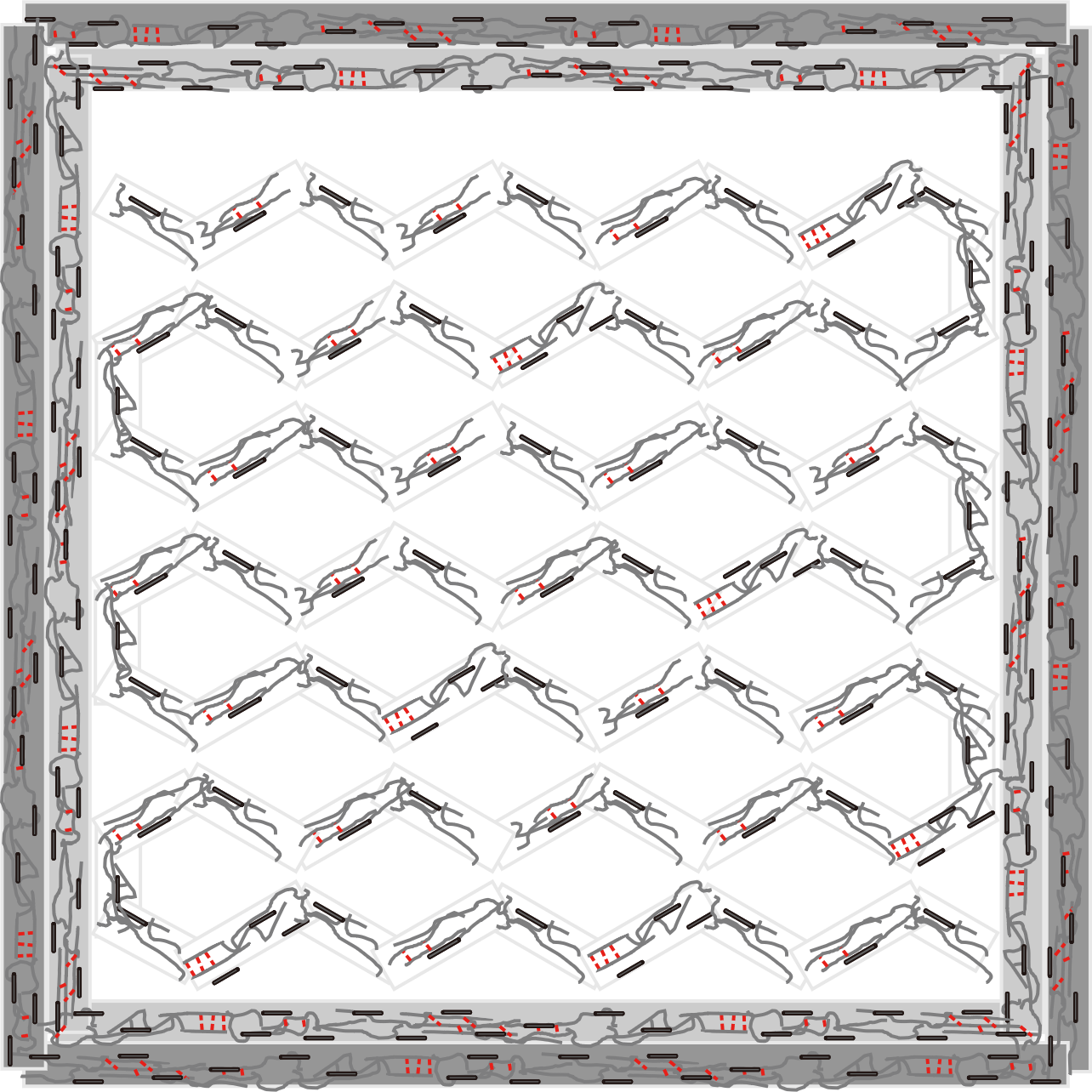** | 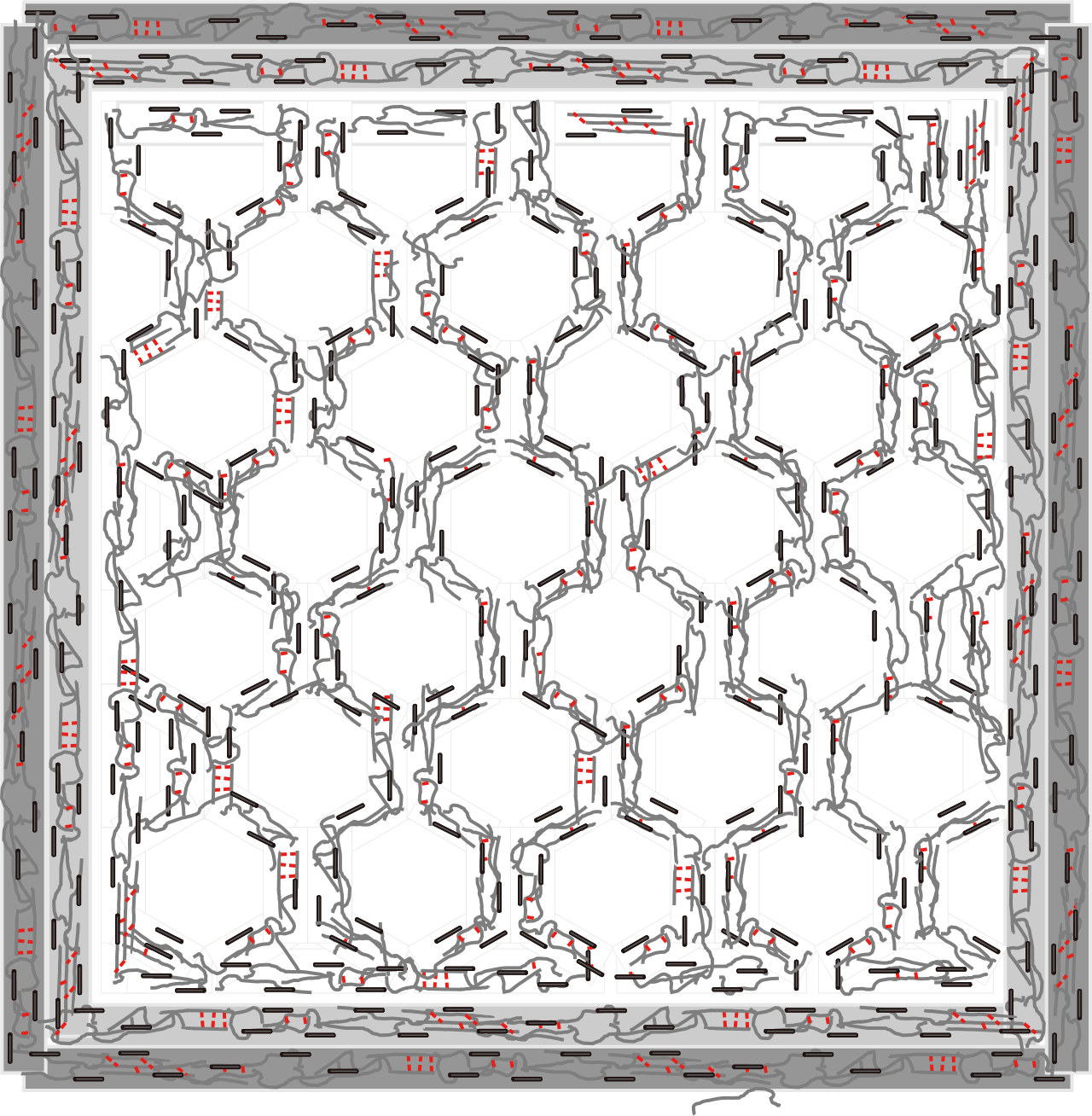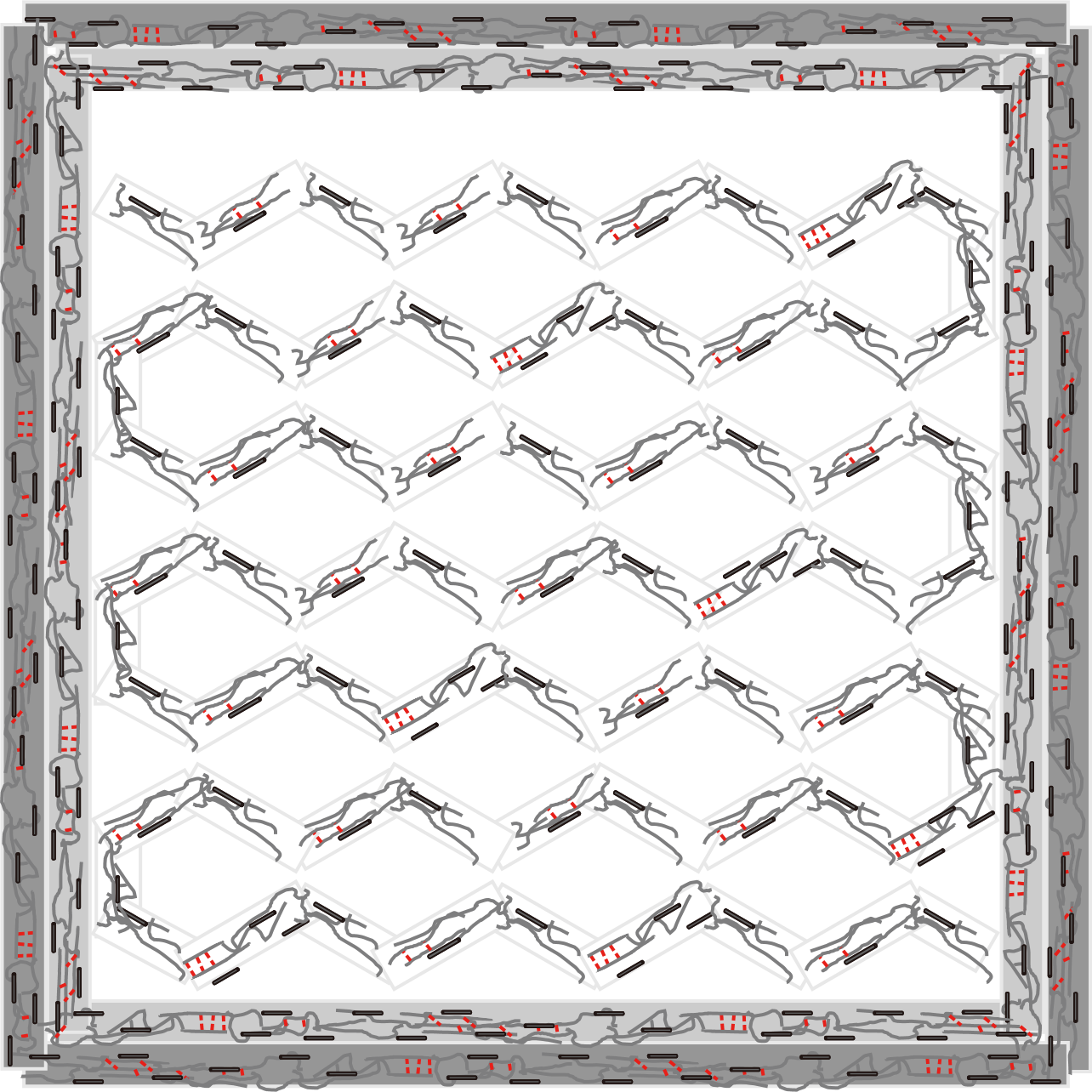 |
